# The channel catfish genome sequence provides insights into the evolution of scale formation in teleosts

**DOI:** 10.1038/ncomms11757

**Published:** 2016-06-02

**Authors:** Zhanjiang Liu, Shikai Liu, Jun Yao, Lisui Bao, Jiaren Zhang, Yun Li, Chen Jiang, Luyang Sun, Ruijia Wang, Yu Zhang, Tao Zhou, Qifan Zeng, Qiang Fu, Sen Gao, Ning Li, Sergey Koren, Yanliang Jiang, Aleksey Zimin, Peng Xu, Adam M. Phillippy, Xin Geng, Lin Song, Fanyue Sun, Chao Li, Xiaozhu Wang, Ailu Chen, Yulin Jin, Zihao Yuan, Yujia Yang, Suxu Tan, Eric Peatman, Jianguo Lu, Zhenkui Qin, Rex Dunham, Zhaoxia Li, Tad Sonstegard, Jianbin Feng, Roy G. Danzmann, Steven Schroeder, Brian Scheffler, Mary V. Duke, Linda Ballard, Huseyin Kucuktas, Ludmilla Kaltenboeck, Haixia Liu, Jonathan Armbruster, Yangjie Xie, Mona L. Kirby, Yi Tian, Mary Elizabeth Flanagan, Weijie Mu, Geoffrey C. Waldbieser

**Affiliations:** 1 The Fish Molecular Genetics and Biotechnology Laboratory, School of Fisheries, Aquaculture, and Aquatic Sciences and Program of Cell and Molecular Biosciences, Auburn University, Auburn, Alabama 36849, USA; 2 National Center for Biodefense Analysis and Countermeasures Center, 110 Thomas Johnson Drive, Frederick, Maryland 21702, USA; 3 Institute for Physical Science and Technology, University of Maryland, College Park, Maryland 20742, USA; 4 Bovine Functional Genomics Laboratory, United States Department of Agriculture, Agricultural Research Service, 10300 Baltimore Avenue, Beltsville, Maryland 20705, USA; 5 Department of Integrative Biology, University of Guelph, Guelph, Ontario, Canada N1G 2W1; 6 USDA, ARS, Genomics and Bioinformatics Research Unit, P.O. Box 38, Stoneville, Mississippi 38776, USA; 7 Department of Biological Sciences, Auburn University, Auburn, Alabama 36849, USA; 8 USDA-ARS Warmwater Aquaculture Research Unit, P.O. Box 38, 141 Experiment Station Road, Stoneville, Mississippi 38776, USA

## Abstract

Catfish represent 12% of teleost or 6.3% of all vertebrate species, and are of enormous economic value. Here we report a high-quality reference genome sequence of channel catfish (*Ictalurus punctatus*), the major aquaculture species in the US. The reference genome sequence was validated by genetic mapping of 54,000 SNPs, and annotated with 26,661 predicted protein-coding genes. Through comparative analysis of genomes and transcriptomes of scaled and scaleless fish and scale regeneration experiments, we address the genomic basis for the most striking physical characteristic of catfish, the evolutionary loss of scales and provide evidence that lack of secretory calcium-binding phosphoproteins accounts for the evolutionary loss of scales in catfish. The channel catfish reference genome sequence, along with two additional genome sequences and transcriptomes of scaled catfishes, provide crucial resources for evolutionary and biological studies. This work also demonstrates the power of comparative subtraction of candidate genes for traits of structural significance.

## Abstract (web summary)

Catfish represent 6.3% of all vertebrate species, and occupy a phylogenetic position close to the common ancestor of bony fish. Liu *et al*. present a reference genome of the channel catfish, and reveal a genomic basis for the evolutionary loss of scales in these species.

## Graphical abstract

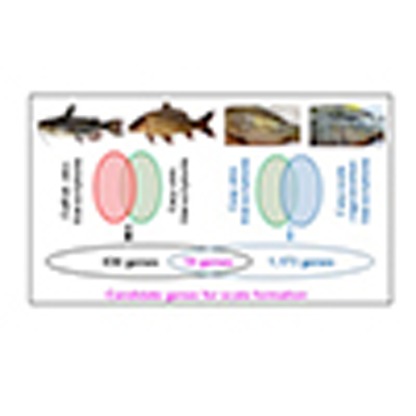

There are roughly 4,100 species in the order of Siluriformes (catfish), which comprise 12% of all fish species and 6.3% of all vertebrate species^1^,^2^,^3^,^4^,^5^,^6^. Catfish are valuable for comparative biological studies because their basal phylogenetic position places them closer to a common fish ancestor than most bony fish (infraclass Teleostei)^7^. Catfish are also valuable worldwide as an important source of dietary protein^4^. During the course of evolution, the catfish lineage lost their scales, but some species reverted to have bony dermal plates covering their skin^8^. The scaled and scaleless catfish offer a natural model for the analysis of genomic basis for the evolution of epidermal appendage formation.

Channel catfish (*Ictalurus punctatus*, family Ictaluridae) is a highly adaptive species as reflected by its broad geographic distribution, tolerance of low water quality and resistance against various infectious agents despite the lack of scales. It is extensively cultured worldwide and is the leading aquaculture species in the United States, accounting for over 60% of US aquaculture production^9^. It has served as a model for comparative immunology, reproductive physiology, and toxicology among ectothermic vertebrates. A high-quality reference genome sequence with annotation of protein-coding genes is essential for understanding of evolution and important biological characteristics such as immune responses to various infectious agents, oxygen metabolism and toxicological processes, as well as for genetic improvement programs^10^,^11^,^12^. Here we report a high-quality reference genome sequence for channel catfish, and its validation and annotation. We also generated draft genome sequences of two South American scaled catfish species, the common pleco (*Pterygoplichthys pardalis*, family Loricariidae) and the southern striped Raphael (*Platydoras armatulus*, family Doradidae). We also generated transcriptomes from skin of the scaled and scaleless (*I. punctatus*) fish and analysed differentially expressed genes during scale regeneration. Through comparative genome and transcriptome analyses during scale regeneration, we provide evidence that implicates secretory calcium-binding phosphoproteins (SCPP) in scale formation in teleost fish.

## Results

### Genome sequencing and assembly

The channel catfish haploid genome contains 29 chromosomes with an estimated 1.0 Gb of DNA^13^,^14^ (Supplementary Fig. 1), and this diploid species is assumed to have undergone the teleost-specific genome duplication (TSGD)^15^,^16^. We generated and assembled the reference genome sequence of channel catfish from a doubled haploid individual^17^ to reduce assembly complexity. Illumina sequence from short insert fragments (400 bp) and mate-paired reads from 3-kb, 8-kb and 34-kb fragments were assembled with MaSuRCA-2.2.0 (ref. 18; Supplementary Table 1). Intrascaffold gaps were filled with Illumina and Illumina-corrected PacBio sequence^19^ (Supplementary Table 2). Further scaffolding was achieved through the use of mate-paired BAC end sequences (BES) and gene transcripts (Supplementary Table 3). The final channel catfish genome assembly included 783 Mb in 34,615 contigs and 9,974 scaffolds, with a contig N50 of 77,200 bp and a scaffold N50 of 7,726,806 bp (Table 1), accounting for ∼78% of the total genome. The assembler collapsed the remaining genome coverage into 159 Mb of repetitive sequence contigs.

The vast majority (96.8%) of the assembled sequence was anchored onto the 29 chromosomes by integration of the locations of SNP markers on the genetic linkage map^20^ with those on the genome sequence scaffolds. Because cytogenetic techniques have not been useful for defining individual chromosomes in this species, we defined the 29 chromosomes based on combined scaffold lengths (Supplementary Table 4). Relative sizes of chromosomes and their linkage groups were positively correlated with an average 218.4 kb per cM.

### Validation of the sequence assembly

**Figure 1:**
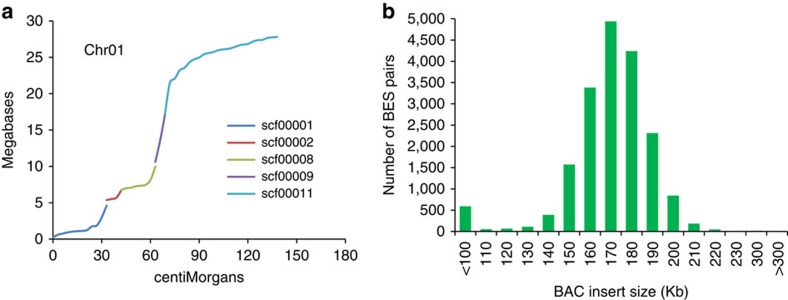
Assessment of assembly accuracy. (**a**) Concordance of SNP marker positions on scaffolds of reference sequence with those on genetic linkage map, with chromosome 1 (linkage group 1=139.5 cM) being shown here. Scaffolds that span <3 cM each were omitted from the graph. (Graphs for all 29 chromosomes are shown in Supplementary Fig. 2). (**b**) Distribution of insert size of BAC clones on the reference genome sequence that was estimated with an average insert size of 161 Kb (ref. 21).

The channel catfish reference genome assembly has a high level of accuracy, continuity and connectivity. The accuracy of the reference genome assembly was first validated by comparing SNP marker positions on the genetic map with their positions in the reference genome sequence. The catfish genetic map contained ∼54,000 SNPs at 31,387 unique SNP locations along 29 linkage groups^20^. The largest 163 scaffolds (totaling 649 Mb) that spanned 3.0–92.6 cM were congruent with the genetic linkage groups (Fig. 1a and Supplementary Fig. 2a–e). The remaining 471 scaffolds that were anchored to chromosomes (totaling 109 Mb) each spanned an average of 0.51 cM in regions of lowered genetic recombination. Second, the mate-paired BAC end sequences were mapped to the reference genome sequence. The patterns of BAC insert sizes on the physical map^21^ and on the reference genome sequence were almost identical (Fig. 1b). Of the 7,471 mate-paired BAC end sequences mapped to a single scaffold of the genome sequence (Supplementary Table 3), only one pair was mapped at a distance >300 kb, which may reflect differences in the genomes of the genome assembly donor and the BAC library donor. Taken together, these data support a high level of accuracy of the reference genome sequence assembly.

The completeness of the genome assembly was revealed as 99.37% of the sequenced bases from 150 individuals (167-fold genome coverage)^22^ aligned along the entire length of the assembly (Supplementary Table 5 and Supplementary Fig. 3). Furthermore, the number of annotated genes compared favourably with the annotations of other teleost genomes (Supplementary Table 6). While the zebrafish (*Danio rerio*) genome assembly^23^ has been regarded as a gold standard among fish genomes, 723 genes included in zebrafish were not found in the channel catfish assembly, but 970 genes found in channel catfish were not found in the zebrafish assembly, suggesting a similar level of completeness.

The assembly achieved a high level of contiguity and connectivity for an Illumina-based assembly of a vertebrate genome. Half of the assembled bases were contained in the 31 largest scaffolds ranging from 7.7 to 22.6 Mb, and 98% of the assembly was contained in only 594 scaffolds of at least 28.0 kb (Table 1). The remaining 2% of the genome assembly was contained in 9,380 smaller scaffolds.

### Genome annotation

The channel catfish genome was predicted to contain 26,661 protein-encoding genes (Supplementary Fig. 4) of which 98.95% were supported by EST or RNA-Seq evidence^24^,^25^,^26^,^27^,^28^. The distribution of these genes among the 29 chromosomes is summarized in Supplementary Table 4 and displayed in Supplementary Fig. 5B. Chromosome orthology between channel catfish and zebrafish was determined with single-copy genes (Supplementary Table 7). The total number of predicted genes in channel catfish is similar to that of zebrafish. When the annotated genes from the assembled genome sequences of channel catfish and zebrafish were compared, a total of 1,010 genes were specific to channel catfish including 143 in-paralogues and 867 single-copy genes; similarly, a total of 931 genes were specific to zebrafish including 243 in-paralogues and 688 single-copy genes (Supplementary Table 8, Supplementary Fig. 6).

### Genomic variation and population history

Previous research indicated a low rate of nucleotide substitution in cartilaginous fish and a high nucleotide substitution rate in zebrafish and stickleback^29^. Alignment of sequence generated from 150 individuals to the catfish reference genome assembly indicated a high rate of nucleotide substitution across the genome (Supplementary Fig. 5C). The rate of one SNP per 93 bp places channel catfish among the most varied vertebrate genomes. Regions with the highest rates of base substitution included 111 genes (Supplementary Data 1), of which 40 were related to immune function. Many of the teleost-specific immune-related genes were located in genomic regions with a high SNP rate (Supplementary Fig. 5D).

**Figure 2:**
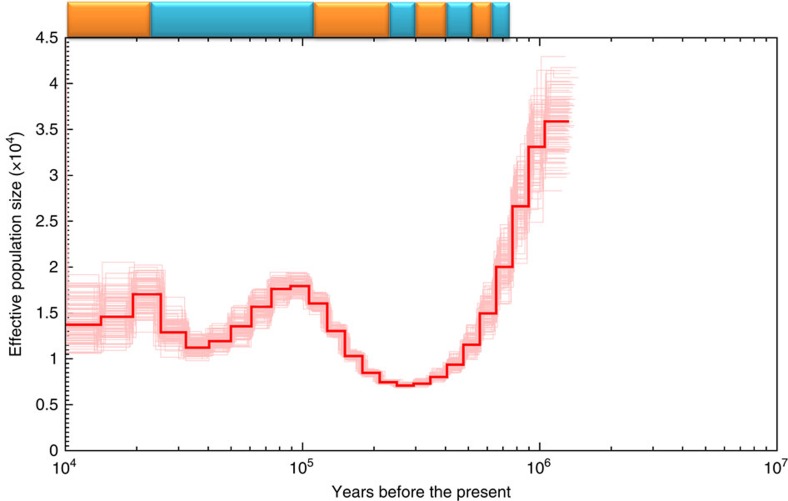
Inference of channel catfish population history. The central bold line represents inferred population size, and the 100 thin curves surrounding the central line are PSMC estimates generated using 100 sequences randomly resampled from the original sequence. The mutation rate of 2.5e−8, adopted from medaka^64^, was used in time scaling. Blue bars at the top of the figure represent glacial periods and orange bars, interglacial periods.

The high level of heterozygous SNPs in channel catfish may reflect a large founder population and perhaps also reflect its recent population expansion. On the basis of the local heterozygous SNP densities, we inferred the channel catfish demographic history using the pairwise sequentially Markovian coalescent model^30^. On the basis of this analysis, channel catfish populations appeared to experience a number of bottlenecks that coincided with glacial activities, with a large population size more than one million years ago, followed by sharp declines after each of the glacial periods, and gradual recovery during interglacial periods (Fig. 2).

### Gene duplications

**Figure 3:**
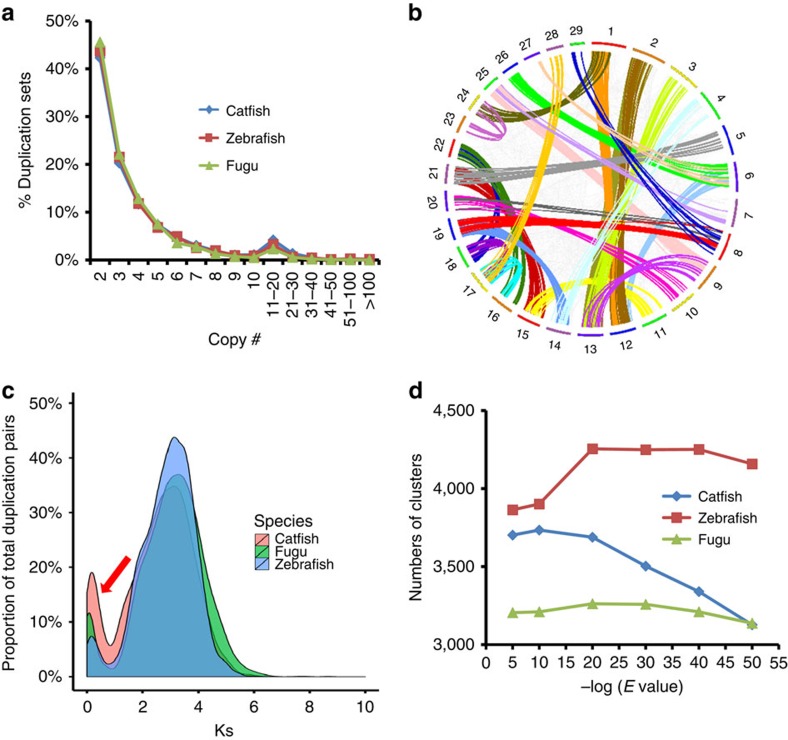
Characteristics of duplicated genes in the channel catfish genome. (**a**) Comparison of gene copy numbers of duplicated genes among catfish (blue), zebrafish (orange) and fugu (green); (**b**) Interchromosomal duplicated channel catfish genes, likely derived from teleost-specific genome duplication. Numbers are chromosomes and coloured lines represent at least 25 duplicated gene pairs; (**c**) Comparison of duplicated genes in channel catfish, fugu and zebrafish, highlighting recent lineage-specific duplication in channel catfish as specified by the red arrow. (**d**) Duplicated gene clusters in zebrafish, fugu and channel catfish as a function of sequence similarities, noting the sharp splitting of duplicated clusters in channel catfish with higher e-values, suggesting a higher proportion of rapidly evolving duplicated gene clusters in the channel catfish genome.

The infraclass teleostei is considered to have evolved ∼340 million years ago^31^ from a common ancestor that underwent the TSGD^16^. We identified 3,688 gene sets in channel catfish that contained two or more copies (Supplementary Data 2), 15.3% fewer than in zebrafish, but 13.1% more than in fugu (Supplementary Table 9). The overall proportion of duplicated gene sets at various copy numbers was similar among channel catfish, fugu and zebrafish (Fig. 3a). The majority of the duplicated gene sets have two copies on different chromosomes, suggesting that they were likely derived from the TSGD (Fig. 3b). The channel catfish genome contained a slightly larger fraction of recently duplicated genes with small Ks values (Fig. 3c). In channel catfish, sequences of a fraction of the duplicated genes (∼400 genes) were highly variable such that they no longer belonged to the same clusters when the cutoff *e*-values were decreased (Fig. 3d). Gene ontology analysis indicated that the genes with greater levels of variability were mostly involved in metabolism of xenobiotic chemicals, immune function, and stress response (Supplementary Data 3), which may reflect selective pressures consistent with the natural habitat of channel catfish as a freshwater bottom feeder.

### Genomic hallmarks of teleost fish

**Figure 4:**
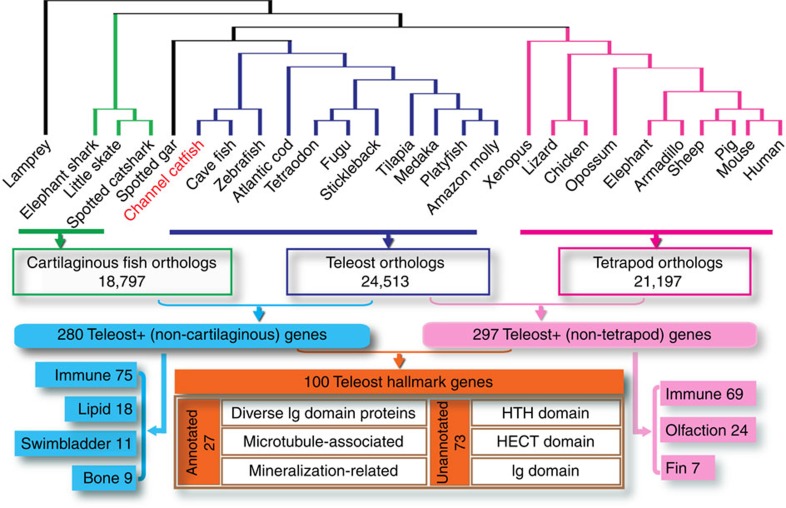
Genomic hallmarks of teleosts. Shown on the top panel are species whose genome sequences were used for the analysis. On the lower panel, comparisons with cartilaginous fish are shown on the left, and comparisons with tetrapods are shown on the right. The genome hallmarks of teleosts are shown in the middle, highlighted by genes for diverse Ig domain-containing proteins, matrix mineralization and microtubule-associated proteins.

We identified 297 genes existing in teleosts but not in tetrapods (Supplementary Data 4). Gene ontology enrichment analysis indicated that the set of genes specific to teleosts and lower vertebrates was enriched for immune function (69 out of 187 known genes), olfactory sensing (24 out of 187), and fin development (7 out of 187) (Fig. 4). A new array of olfactory receptors, specifically the ζ family of olfactory receptors, evolved and was uniquely amplified among teleosts^32^. In addition, a large set of immunoglobulin (Ig) domain-containing genes were specifically evolved in the teleost lineage^33^. We also identified 280 genes that were not present in cartilaginous fish or jawless species but were present in teleost fish (Supplementary Data 5). These genes were enriched for function in bone development, immune function, swim bladder development and lipid metabolism. During the course of evolution, a set of genes involved in bone development, including those encoding the SCPP involved in matrix mineralization, were lost in cartilaginous fish^34^ but duplicated in the teleost lineage, providing a genomic foundation for the development of the endoskeleton. The swim bladder is unique in teleosts, and a set of 11 genes demonstrated high levels of expression in swim bladder tissue (Supplementary Table 10). A few genes involved in lipid metabolism were unique in teleosts (Supplementary Data 5), and contained phospholipase domains that are linked to taste signal transduction in fish^35^,^36^. Apolipoproteins were highly expressed in mucus associated with skin as early response molecules to infection^37^. Thus, in the present research, we put forward a set of 100 genes identified as hallmarks of teleost genomes that are absent in tetrapods and cartilaginous fish (Supplementary Data 6).

### Genomic basis for scale formation

**Figure 5:**
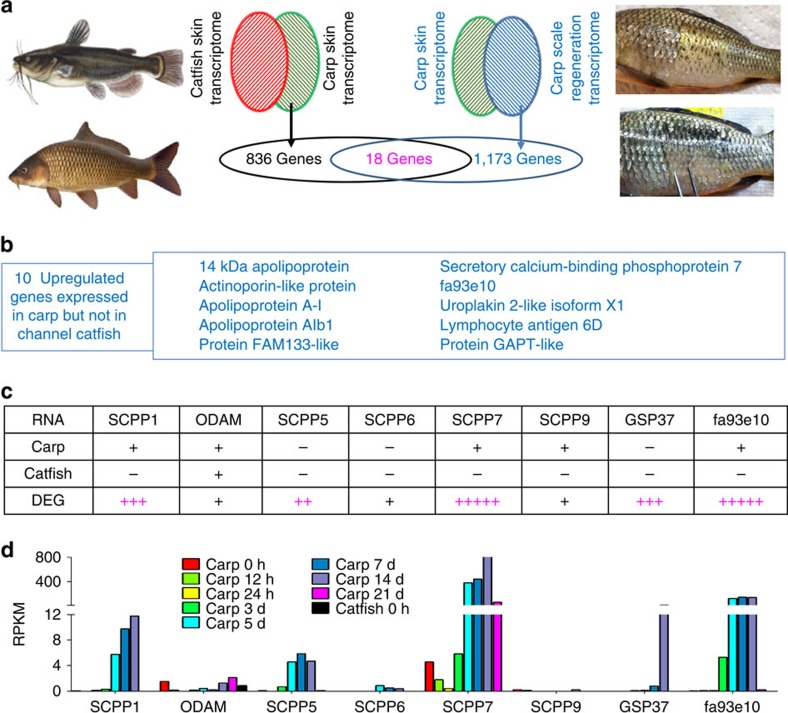
Comparative subtraction analysis of transcriptomes. (**a**) Interspecific comparative transcriptome analysis: the channel catfish skin transcriptome and the carp skin transcriptome was comparatively subtracted, leading to the identification of 836 genes expressed only in the carp skin but not in the channel catfish skin (left); similarly, the carp skin transcriptomes before scale regeneration and during scale regeneration were comparatively subtracted, leading to the identification of 1,173 differentially expressed genes during scale regeneration. The pool of the 836 genes and 1,173 genes shared (intersection) a total of 18 genes including 13 known genes and five uncharacterized genes. (**b**) A list of the 10 upregulated genes identified from the shared intersection of the genes expressed in carp but not in channel catfish skin and those differentially expressed during scale regeneration. (**c**) A summary of SCPP expression in the carp skin (Carp), catfish skin (Catfish) and during scale regeneration (DEG). Plus (+) indicates expression and minus (−) indicates no expression. Multiple pluses indicate degree of induced expression, and purple colour indicated that they were significantly upregulated. (**d**) SCPP expression during the course of scale regeneration, with their expression levels expressed as reads per kilobase of exon per million mapped reads (RPKM) on the *y* axis, genes indicated on the *x* axis, and time points expressed in bars of different colours.

One of the most visible characteristics of most catfish species is the lack of scales. The availability of the whole-genome sequence from scaleless channel catfish enabled a search for the genomic basis for the scaleless phenotype. We first determined whether genes known to cause scale loss when mutated are present in the channel catfish genome. Sequence analysis indicated that ectodysplasin A (*EDA*), EDA receptor (*EDAR*), fibroblast growth factor receptor (*FGFR*), lymphoid enhancer-binding factor 1 (*LEF1*), and T-cell-specific transcription factor 7 (*TCF7*)^38^,^39^,^40^ were all present and expressed in channel catfish (Supplementary Figs 7–11). We further conducted comparative transcriptome analysis between the scaled and scaleless fishes. Our experiments indicated that scale regeneration was not feasible with bony dermal plate-type of scales found in the armored catfish; therefore, we conducted transcriptome analysis between the scaled common carp and scaleless channel catfish, using skin tissues from which scales are derived. The interspecific transcriptome analysis (Fig. 5a) revealed 836 genes expressed in common carp but not in channel catfish (Supplementary Data 7). We hypothesized that genes important for scale formation should be differentially expressed. Therefore, we conducted scale regeneration experiments and analysed gene expression at various time points after scale removal. Compared with the controls, 1,173 genes were found to be differentially expressed (Supplementary Data 8) during scale regeneration. Cross subtraction of the interspecific skin transcriptomes and the differentially expressed genes during scale regeneration revealed 13 annotated and 5 unannotated genes. Of the 13 annotated genes, 10 were upregulated and three were downregulated during scale development (Fig. 5b). The most prominent of the upregulated genes were apolipoprotein and SCPP genes. Apolipoproteins are known to promote fat efflux^41^ and have been reported to be involved in scale development in zebrafish^42^,^43^, suggesting their role in development of mineralized tissues. SCPP genes arose from *SPARCL1* through gene duplication^44^,^45^, and are involved in skeletal and dental tissue mineralization^46^,^47^,^48^,^49^,^50^,^51^. Although the involvement in scale development was not previously known with the exception of *GSP37*; the SCPPs were not found in the cartilaginous elephant shark genome^29^,^34^. During carp scale regeneration, expression of *ODAM*, *SCPP6* and *SCPP9* was not elevated. However, *SCPP7* and *fa93e10* were drastically induced (Fig. 5c,d). In addition, *SCPP1*, *SCPP5* and *GSP37* were also significantly induced. The timing of expression of the SCPP genes corresponded well with scale regeneration^52^, beginning on day 5 throughout 3 weeks after scale removal (Fig. 5d). The results point to the products of these 18 differentially expressed genes, especially the SCPP genes, as candidates in the scale formation pathway.

### Some SCPP genes were lost in the channel catfish genome

**Figure 6:**
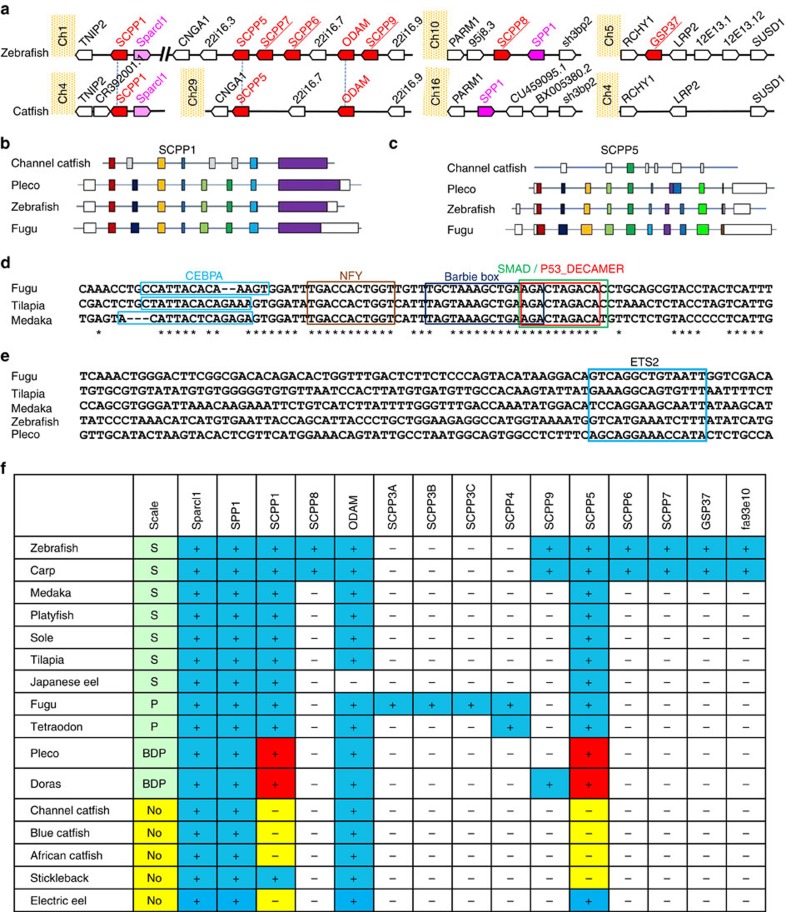
Status of SCPP genes in channel catfish and various other fishes. (**a**) Conserved syntenic regions of catfish and zebrafish containing SCPP genes were identified. In zebrafish, the SCPP genes were located on chromosomes 1, 10 and 5, whereas in channel catfish the SCPP genes were on chromosomes 4, 29 and 16. Note that SCPP7, SCPP6, SCPP9, SCPP8 and GSP37 are missing from the channel catfish genome (underlined). (**b**) Comparison of SCPP1 genes in channel catfish, pleco, zebrafish and fugu, noting that three exons (2, 5 and 6; light shaded) the channel catfish SCPP1 gene was mutated or deleted. (**c**) Comparison of SCPP5 genes in channel catfish, pleco, zebrafish and fugu, noting that only a small segment (green box) of SCPP5 homologous sequences was present as remnant, and most of its coding sequences are deleted. (**d**) Alignment of the sequences in the promoter regions of SCPP5 in fugu, tilapia and medaka, noting the highly conserved sequences in the transcriptional factor binding sites, which are entirely lost in the channel catfish gene (not shown). (**e**) Alignment of the sequences in the promoter regions of SCPP1 in fugu, tilapia, medaka, zebrafish and pleco, noting the highly conserved ETS2 transcriptional factor binding sites, which are lost in the channel catfish gene (not shown). (**f**) Status of SCPP genes in various teleost species in relation to their scale status (BDP, bony dermal plates; No, no scales; S, scaled; P, protuberance). Pluses (+) in blue shading indicate presence of SCPP genes, and minuses (−) indicate absence of the SCPP genes; yellow shading highlights specific absence of the SCPP genes in scaleless fishes, and red shading indicates presence of SCPP1 and SCPP5 in scaled catfish common pleco (pleco) and southern striped Raphael (doras).

We then determined the status of SCPP genes in channel catfish. The channel catfish genome contains only three SCPP genes: *SPARCL1*, *ODAM* and *SPP1*. Analysis of gene synteny between the catfish and zebrafish genomes revealed the *SCPP6*, *SCPP7*, *SCPP8*, *SCPP9*, *GSP37* and *fa93e10* genes were absent in the channel catfish genome (Fig. 6a). Sequences similar to *SCPP1* and *SCPP5* were identified in the catfish genome in the conserved syntenic regions; however, three coding exons (2, 5 and 6) of *SCPP1* could not be identified in the channel catfish sequence (Fig. 6b), only a short remnant of *SCPP5* involving coding exon 5 was found in the catfish genome (Fig. 6c), the transcriptional factor binding sites within the promoter regions of both genes were missing (Fig. 6d,e), and no transcripts were identified for either gene in the transcriptome of channel catfish.

### SCPP genes in scaled and scaleless fishes

We then examined the genomic status of the SCPP genes in fish species that have or lack scales (Fig. 6f). *SCPP5* and *SCPP1* were most relevant to scale formation as they were present in all scaled fish but one or both were absent from all scaleless fish. Neither SCPP1 nor SCPP5 were present in the genomes of all examined catfish species. With the three-spine stickleback, a scaleless fish, SCPP5 was absent while SCPP1 was present in its genome; with electric eel, another scaleless fish, SCPP5 was present but SCPP1 was absent from its genome, suggesting that loss of SCPP1 or SCPP5, or both, would cause the scaleless phenotype. The three-spine stickleback does not have scales but has bony plates along the sides of the body, although there is a subspecies (*G. aculeatus williamsonii*) that is unarmored. While *SPP1*, *SPARCL1* and *ODAM* may be involved in developmental pathways leading to scale formation, their presence in both scaled and scaleless fish suggests their roles do not determine the scaled phenotype.

The number of SCPP genes varied among the analysed species. *SCPP6*, *SCPP7*, *SCPP8*, *SCPP9*, *GSP37* and *fa93e10* may be derived from lineage-specific gene duplications as they are present only in certain fish such as carp and zebrafish; similarly, *SCPP3A*, *SCPP3B*, *SCPP3C* and *SCPP4* could be lineage-specific duplications in fugu and Tetraodon; while they may be involved in scale development and scale type, their absence does not appear to account for the lack of scales in catfish because they are also absent in the scaled medaka, platyfish, sole and tilapia (Fig. 6f).

### Scaled catfishes harbour and express *SCPP1* and *SCPP5*

**Figure 7:**
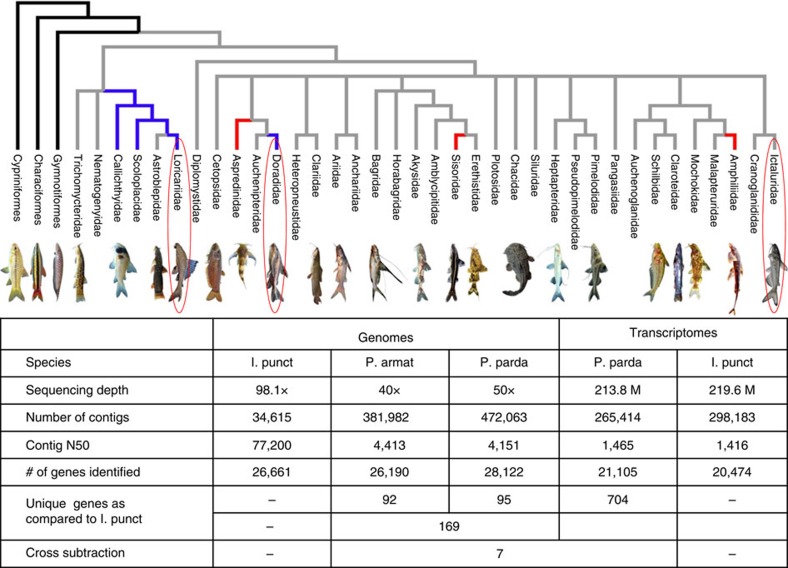
Generation and analysis of genome and transcriptome sequences from scaled catfishes. Scales represent the primitive condition in the Otophysi (black branches), with catfishes lacking scales (grey branches). Bony plates have evolved several times within catfishes and include all members of the family (blue branches) or just some members of a family (red branches). It is unknown whether the plated condition within callicthyids, scoloplacids and loricariids is homologous. Phylogeny simplified from Sullivan *et al*.^6^. Photos by J.W. Armbruster, N.K. Lujan, M.H. Sabaj-Perez, S. Smith, K. Luckenbill, H.H. Ng, Z. Randall and L.M. Page. I. puntat, *Ictaluras punctatus*; P. armat, *Platydoras armatulus*; P. parda, *Pterygoplichthys pardalis*. The genes listed with ‘cross subtraction' are the number of genes that were found and expressed in the genome of scale catfishes, but not present in the genome of scaleless channel catfish.

While most catfish species do not have scales, some catfish species have scales made of bony dermal plates. Our hypothesis that SCPP genes are involved in scale formation would predict the expression of SCPP genes in the skin of scaled catfishes. Therefore, we generated draft genome sequences from two scaled (armored) catfish species, *Pterygoplichthys pardalis* and *Platydoras armatulus* to examine the status of SCPP genes and analysed the transcriptome of *P. armatulus* skin (Fig. 7) to measure the expression of SCPP genes. Comparative genome subtraction revealed 169 genes that were present in the armored catfish species but absent from channel catfish, including *SCPP1* and *SCPP5* (Supplementary Data 9). Sequence analysis demonstrated the *SCPP1* and *SCPP5* open reading frames were intact in both armored catfish. Similarly, subtractive analysis of *P. armatulus* and channel catfish skin transcriptomes revealed 704 genes that are expressed in the skin of scaled *P. armatulus* but not in scaleless channel catfish (Supplementary Data 10). Subtraction of genes and transcripts found in both species revealed seven genes differentially expressed in *P. armatulus* skin, including the transcripts of *SCPP1* and *SCPP5* (Fig. 7; Supplementary Table 11). Taken together, these results implicated *SCPP1* and *SCPP5* in scale formation in the armored catfish, and the lack of these two genes in channel catfish is related to the lack of scales.

### Expression of SCPP genes in various ossified tissues

**Figure 8:**
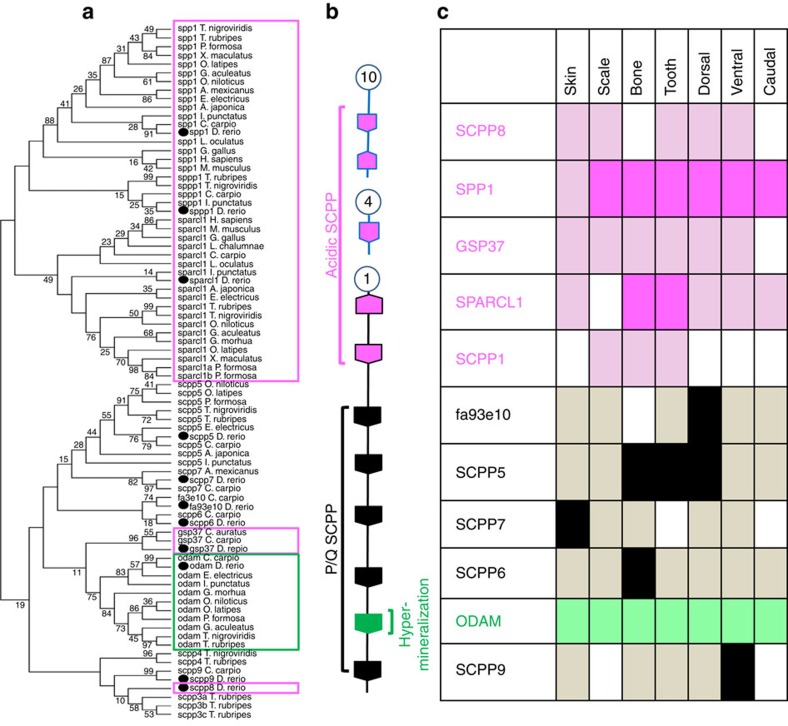
Phylogenetic analysis and expression of SCPP in zebrafish. (**a**) Phylogenetic analysis of SCPP genes. Acidic SCPP genes are indicated by hot pink box, and the hypermineralization ODAM is indicated by the green box, and the remaining are P/Q type SCPP genes. (**b**) Chromosome location and types of SCPP genes indicated with different colour (hot pink, acidic SCPP genes); black, P/Q type SCPP genes and green, hypermineralization SCPP. (**c**) Summary of expression patterns of the SCPP genes in scale, bone, tooth and fins. Boxes filled with solid hot pink colour represent high expression of the acidic SCPP in that tissue; boxes filled with solid black colour represent high expression of the P/Q SCPP in that tissue; boxes filled with light pink or grey colour are acidic or P/Q type, respectively, SCPP genes that are expressed at low levels in the tissue; and white boxes represent no expression of the specific SCPP genes in the tissues. Note that SPARCL1 was highly expressed only in bone and tooth. SCPP7 was highly expressed in the skin; SCPP6 was highly expressed in the bone; SCPP5 was highly expressed in bone, tooth and dorsal fin; and SCPP9 and fa93e10 was most highly expressed in the ventral fin and dorsal fin, respectively. ODAM was expressed at intermediate levels in all tested tissues.

The roles of SCPP genes in scale formation were previously unknown. Among all the known SCPP genes, only *GSP37* was reported as a matrix protein extracted from goldfish scales. Phylogenetic and syntenic analysis indicated that *SCPP5* and *SCPP7*, both P/Q-rich SCPPs, are likely paralogues; *SCPP6*, *SCPP9* and *ODAM*, all P/Q-rich SCPPs, are likely paralogues (Fig. 8a,b); and *SPP1*, *SCPP8* and *GSP37*, all acidic SCPP, are likely paralogues, derived from a duplication event.

To gain insights into SCPP expression and function in scale formation, tissue expression of SCPP genes was analysed by RT–PCR in zebrafish. Although the SCPP genes were expressed in multiple tissues (Fig. 8c), we observed patterns in the most highly expressed SCPP genes: the acidic *SPARCL1* was most highly expressed in tissues with the highest levels of ossification, bone and tooth, while the acidic *SPP1* was highly expressed in all ossified tissues. In contrast, expression of P/Q type SCPPs varied greatly among ossified tissues. These expression patterns indicated a continuum of SCPP expression and tissue mineralization^40^,^45^,^50^, with the hard bone and tooth highly expressing the acidic SCPPs and scales and fins highly expressing the P/Q type SCPPs as well as the acidic *SPP1*.

## Discussion

In this work, we sequenced and assembled the reference genome sequence from channel catfish, *Ictalurus punctatus*. This is the first reference genome sequence from catfish, a group including more than 4,100 species, and will be valuable for biological and evolutionary research and in facilitating selective breeding of catfish broodstock for aquaculture production. The reference genome achieved high levels of completeness, contiguity, connectivity and accuracy. Among all sequenced fish genomes, the contig and scaffold N50 statistics of the channel catfish assembly were second only to those of stickleback, whose genome was sequenced by using Sanger sequencing^53^. The vast majority of the assembled sequence (96.8%) was anchored to the 29 haploid chromosomes, the highest among all sequenced fish genomes. Technological advances will continue to make *de novo* assembly of vertebrate genomes easier and more affordable, but it is important, and challenging, to produce accurate *de novo* assemblies. The use of a homozygous, doubled haploid genomic template, PCR-free Illumina sequencing libraries, long mate-paired sequences and PacBio long read sequences were combined to produce a highly contiguous assembly. However, the identification and correction of several hundred scaffold mis-assemblies in the initial assembly by comparison to the high-density genetic map demonstrated the importance of an independent genomic resource in validating and improving the connectivity and accuracy of the *de novo* assembly.

An important discovery in the channel catfish genome assembly was the identification of a larger number of lineage-specific, recently duplicated genes (Fig. 3). Although the overall level and pattern of gene duplications of channel catfish is similar to that of other fish species such as zebrafish and fugu, a prominent increase in recently duplicated genes (Ks<1) was present with channel catfish. Gene ontology analysis indicated that many of these genes were involved in metabolism of xenobiotic chemicals, immune function, and stress responses. Analysis of genomic variation revealed more than 8.3 million SNPs across the channel catfish genome, although their distribution varies along its 29 chromosomes (Supplementary Fig. 5). Such an SNP rate made catfish one of the species with most highly variable genome among vertebrate species. It is notable that highly variable genomic regions correlate with positions of many of the teleost-specific genes (Supplementary Fig. 5), for example, the genomic regions with the highest SNP rates contained an enriched set of immune-related, Ig domain-containing protein genes. The gene duplications and high level of SNP variation may have contributed to evolutionary adaptation of this species to aquatic environments with lowered water quality and to immune protection in the absence of scales.

Although SCPP genes were previously known to be involved in mineralization of bones and teeth^29^,^40^,^44^,^45^,^46^,^47^,^48^,^49^,^50^,^51^, only *GSP37* was known to be a matrix protein extracted from the goldfish scales^54^. We report here, for the first time, the involvement of all SCPPs in scale formation. Our findings also provide strong evidence that the loss of SCPP genes accounted, at least in part, for the evolutionary lack of scales. Here genomic analysis of scaled and scaleless catfishes as natural models was used for the identification of genes involved in scale formation. Genome comparison and transcriptome subtraction, as demonstrated here, was effective for discovering candidate genes for phenotypes of interest. Gene editing systems are not yet available for scaled catfish, but could be used in zebrafish to validate the roles of SCPP genes in scale formation in future studies. However, the redundant copies of SCPP genes, with some, for example, *SCPP1*, *SCPP5*, *SCPP7* and *fa93e10*, having almost identical expression patterns during scale regeneration (Fig. 5d), may complicate functional analyses.

## Methods

### Generation of genome sequences

A doubled haploid channel catfish individual^17^ was used as template for sequencing. Genomic DNA was isolated from blood cells. Genomic DNA was sheared with a HydroShear instrument (Digilab, Inc., Marlborough, MA) using the regular chamber and set at shear code 13 for 20 cycles for 3-kb fragments and shear code 16 for 20 cycles for 8-kb fragments. Fragments were separated by field inversion agarose gel electrophoresis, isolated with a Whatman Elutrap elution system, and concentrated in a Microcon-50 (EMD Millipore, Billerica, MA). For fosmid library production, genomic DNA was sheared in the large chamber at shear code 15 for 30 cycles and purified as above, and the DNA was cloned into the pCC2FOS vector (Epicentre, Madison, WI) using the CopyControl HTP Fosmid Library Production Kit.

For Illumina sequencing, an Illumina TruSeq PCR-Free LT library was produced from Covaris-sheared genomic DNA of 400-bp and sequenced on the NextSeq500 platform. An Illumina Mate Pair Library Prep Kit was used to produce 3-kb and 8-kb fragment libraries, and these fragments were sequenced on an Illumina GAIIx and HiSeq platform, respectively. Paired-end sequences were produced from pooled fosmids, and sequenced on the Illumina GAIIx platform. Pacific Biosciences long read sequence was produced from 8–10 kb genomic DNA fragments with C2 chemistry (Expression Analysis, Durham, NC). The sequences were error-corrected using the pacbioToCA algorithm within Whole Genome Shotgun assembler v7.0 (ref. 55).

### Sequence assembly

Illumina sequencing data was assembled using MaSuRCA-v2.2.0 (ref. 18). Trusted k-mers were identified in the short insert library using *jellyfish*-2.0 (ref. 56), and reads were trimmed using quorum. The MaSuRCA pipeline normalized redundant mate-paired and fosmid end paired sequences and trimmed chimeric reads, then all reads were assembled to produce superReads. SuperReads, paired-end reads that linked superReads, and paired reads from the jumping libraries were submitted to Celera Assembler utilizing a maximal scaffolding error rate of 10%. After initial assembly, gaps in the primary scaffolds were closed by manually iterating the final gap-closing process in the MaSuRCA-2.2.0 pipeline. Quorum-corrected Illumina reads were queried against non-repetitive sequences flanking the scaffold gaps and the output was stored before filling the gaps. This process was repeated with full-length Illumina-corrected PacBio sequences and also with PacBio sequences that were shredded to 300, 500, 750, 1,000, 1,500, 2,000 or 3,000 bp lengths with 20% sequence overlap. The resulting PacBio gap-fill output was filtered to remove sequences that were present in the Illumina gap-fill output. Filtered PacBio output from all iterations was normalized, combined with the Illumina output, and processed through the MaSuRCA gap-closing pipeline.

After initial assembly, SNP markers on the genetic map^20^ were aligned to the scaffolds using MUMmer to identify inconsistencies between the scaffolds and the genetic map. Illumina and corrected PacBio reads were remapped to scaffolds with Burroughs–Wheeler Aligner and visualized with Integrated Genomics Viewer (IGV) to guide manual breaking of scaffolds that contained multiple unmatched mates. Scaffolding errors occurred mainly in superReads that were incorrectly assembled at long mono-, di- or tri-nucleotide repeats at the end of the Illumina reads. These scaffolds were manually broken and rejoined by overlap sequences. After these initial stages of assembly, the gaps of unknown sizes within the scaffold sequences were replaced by 100 N's before submission to GenBank.

Additional scaffolding was conducted using mate-paired BES^57^,^58^. The BES were aligned with repeat-masked scaffolds using BLASTN with a minimum 95% sequence identity over 70% of the BES length, and sequences with multiple matches were discarded. We required a minimum of two pairs of BES for manual scaffolding with proper orientation. In addition, ESTs and RNA-Seq assembled transcripts were used to bring scaffolds together if the parts of a single gene were located in separate scaffolds. Additional scaffolding using ESTs and RNA-Seq transcripts^24^,^25^ was conducted using L_RNA-Scaffolder^59^. Chromosome level scaffolds were assembled based on the genetic linkage map^20^. Adjacent scaffolds along a chromosome were manually joined with a string of 100 ‘N''s to represent the gaps between the two adjacent scaffolds.

### Assessment and validation of the sequence assembly

The completeness of the genome-sequence assembly was assessed by aligning the assembly of the 167X independently generated genomic sequences from 150 individuals to the reference genome assembly using MUMmer3.23 (http://mummer.sourceforge.net/) with default settings. The short reads from the 150 individuals were assembled using ABySS^60^. In addition to genome sequence alignments, the number of genes included in the sequence assembly was used as a parameter for assessing the completeness. Channel catfish genes were compared with those of 12 teleost species whose whole genome has been sequenced (Supplementary Table 6). Protein-coding genes of these species were retrieved from Ensembl (version 78), with exception of the genes of *Cynoglossus semilaevis* (Cse_v1.0), which were retrieved from NCBI. For genes with multiple splicing variants, the longest variant was used. Only genes encoding proteins of >30 amino acids were used in the analysis. First, all proteins from the channel catfish and the 12 other species were combined in an all-versus-all BLASTP comparison with maximal e-value of 1e−5. Clusters of orthologous groups among these 13 species were identified using SiLiX (ref. 61) with minimum identity of 30% and minimal sequence overlap of 50%. Comparison of gene content was conducted using BLASTP analysis with a maximal e-value of 1e−5. The predicted protein sequences of channel catfish were queried against protein sequences of each of the 12 teleost species, separately. If all members of an orthologous group of catfish proteins had no match in a given species, then the gene was deemed present in channel catfish (Catfish+) but absent from the species under comparison. Similarly, the ‘Catfish−' genes were identified through reciprocal BLASTP comparisons of protein sequences of the other 12 species against channel catfish. Because the zebrafish genome has been considered ‘complete', a similar analysis was conducted to generate ‘Zebrafish+' and ‘Zebrafish−' genes for comparison. The correctness/accuracy of the sequence assembly was assessed by comparing SNP marker positions on the genetic map versus those on the genomic sequence scaffolds using positions determined as above with MUMmer. In addition, the mate-paired BES that aligned within a single scaffold were used to assess assembly accuracy.

### Genome annotation

AUGUSTUS and FGENESH (http://www.softberry.com) were used for genome annotation (Supplementary Fig. 4). Gene model parameters for AUGUSTUS were trained from conserved genes from vertebrate species using CEGMA. Then, the amino-acid sequences predicted from both software were used as queries against the Uniprot and NCBI non-redundant databases. For those predicted coding regions with no blast hits, but containing >100 amino acids, both Pfam A and B databases were used to search functional domains. The tandem duplicated genes were identified using MCScanX. Only the longest protein of each subgroup was kept to represent the corresponding gene. The final catfish genome annotation included genes with names derived from BLAST analysis and genes with domain names derived from Pfam analysis. The visualization of gene density within 1 Mb of the whole genome was generated using Circos^62^.

### Comparative genome analysis

The catfish genome sequence was compared with that of zebrafish to determine chromosome orthology. The homologous chromosomes were determined as the chromosomes with maximal gene homology (Supplementary Table 9). First, the catfish and zebrafish proteins were combined and an all-versus-all BLASTP with a maximal e-value of 1e−5. The OrthoMCL pipeline was used to define protein similarities with a minimum 50% length coverage and maximal e-value of 1e−5. MCL generated the potential orthologue relationships between catfish and zebrafish with the inflation parameter set at 1.5. To obtain the species-specific genes, a further round of BLASTP was performed in which genes not included in the orthologue groups were queried against the genes in the orthologue groups within the same species, with a maximal e-value of 1e−10. A reciprocal BLASTP with maximal e-value of 1e−5 was used to query genes with no hits from previous steps. The orthologues were consequently categorized in nine classes: one to one, one to two, one to many, two to one, two to two, two to many, many to one, many to two and many to many. Genes not in the orthologue groups were identified as in-paralogues or single-copy species-specific genes using SiLiX (ref. 61; Supplementary Table 8).

### Analysis of genome variations

For SNP analysis, we used the 167 × genome equivalent of Illumina short reads from resequencing of 150 individuals^22^ and RNA-Seq reads from 602 individuals. STAR Aligner (version 2.4.0)^63^ was used to align the short reads to the reference genome sequence. SNPs were identified using the criteria of base quality ≥20, mapping quality ≥20, minor-allele frequency ≥5%, and sequencing depth coverage ≥6.

Mapping of the SNPs to the reference genome sequence was conducted by aligning sequences of 35 bp from each side of the SNP locus. SNPs with flanking regions that contained more than one alignment were removed from analysis. Sequences mapped to low complexity and repetitive regions were also removed. The number of SNPs within 100 Kb bins was calculated to estimate the overall distribution of SNPs. Of each chromosome, the bin with the highest SNP rate was regarded as the hot spot of genomic variations. Genes contained within these hot spots were identified by BLAST analysis.

### Inference of demographic history

Demographic history was reconstructed based on a Hidden Markov Model (HMM) approach using PSMC^30^. Briefly, the genomic sequences generated from each diploid channel catfish were aligned to the channel catfish reference assembly using BWA mem (version 0.7.12-r1039) with default settings. The consensus sequences were called using SAMtools (version 0.1.19). The ‘fq2psmcfa' tool was used to create the input file for PSMC modelling, with the option -q20. The consensus sequences were divided into non-overlapping 100 bp bins, with a bin scored as heterozygous if there was a heterozygote in the bin, otherwise it was scored as homozygous. The resultant bin sequences were used as the input for the PSMC estimates using ‘psmc' with the options -N25 -t15 -r5 -p ‘4+25*2+4+6'. The reconstructed population history was plotted using ‘psmc_plot.pl' with the options -u 2.5e-08 -g 7. Because plotting the results required input of generation time (-g 7) and mutation rate (-u 2.5e-08), generation time was calculated as: g=a+[s/(1-s)], where s is the expected adult survival rate which is recorded as 80% in channel catfish, and a is the sexual maturation age that is 3-year for channel catfish. Therefore, the generation time used in this PSMC model was determined as: g=3+[0.80/(1-0.80)]=7. The mutation rate was set following the rate described in a previous study in medaka^64^.

### Analysis of duplicated genes

The channel catfish, zebrafish, and fugu genomes were compared with identify duplicated genes. The optimal sequence similarity threshold was first determined by self-BLASTP searches in each species with e-values from 1e−5 to 1e−50 and gene clusters were produced using MCL. The default parameter of MCL was used [‘mul (0.4343), ceil (200)'] to generate the gene clusters through the ‘mcl' and ‘mcxdump' modules. The initial gene clustering results of each e-value in catfish, zebrafish and fugu were first plotted to determine the optimal level of sequence similarities for further analysis.

The genomic distribution of duplicated catfish genes was determined using the gff file of the genome annotation results. The duplicated genes was classified into three categories: (1) Tandem duplications, if located on the same scaffold within 10 kb; (2) Intrachromosomal duplication (non-tandem), if located on different scaffolds but on the same chromosome; 3) Interchromosomal duplications, if located on different chromosomes. The synonymous substitution rates of duplicated genes was calculated using the KaKs Calculator v2.0 (ref. 65) utilizing a maximum-likelihood model averaging algorithm. The gene duplications with Ks smaller than 1.0 were defined as the recent duplication. The gene ontology enrichment analysis was performed on candidate recently duplicated genes using Ontologizer 2.0 (http://compbio.charite.de/contao/index.php/ontologizer2.html). Enrichment analysis was conducted using the children-union method with Bonferroni correction.

To analyse the Ohnologs of the catfish duplicated genes, an all-against-all comparison between catfish protein sequences and human protein sequences was performed using BLASTP with maximum e-value of 1e−10. The best-matched human gene was recorded for each catfish gene. If human genes matched multiple catfish genes, then the two best matches were defined as paralogues associated with a human gene in catfish. We then paired Ohnologous chromosomes according to the number of paralogues between two chromosomes through Circos 0.67 (ref. 62).

### Identification of genomic hallmarks of teleosts

We used a strategy as reported previously^29^ to identify genomic hallmarks of teleost fish. First, a ‘teleost ortholog core set' was obtained by comparing zebrafish genes with genes from each of the other 10 teleosts: channel catfish, medaka, platyfish, tetraodon, fugu, stickleback, tilapia, Atlantic cod, Amazon molly and cavefish. The zebrafish orthologues of channel catfish genes were generated using the program Inparanoid^66^. The zebrafish orthologues for genes of other species were retrieved from Ensembl v78. The ‘teleost ortholog core set' was prepared from the union set of zebrafish orthologues with all other teleost fish. Second, a ‘tetrapod ortholog core set' was obtained in a similar manner by comparing zebrafish genes with genes from each of the 10 tetrapod species: human, mouse, sheep, pig, elephant, armadillo, opossum, chicken, lizard and frog. The initial set of ‘teleost-specific' genes, that is, genes absent in tetrapods, were obtained by comparing the ‘teleost orthologue core set' with the ‘tetrapod orthologue core set'. The identified genes were further refined by BLAST searches against the nr database (NCBI) to exclude genes that matched tetrapod protein sequences. In addition, the identified ‘teleost-specific' genes required presence in zebrafish and at least two other teleost species. The identified genes were annotated by gene ontology analysis and gene ontology enrichment analysis with their functions identified in research literature and other databases such as GeneCards (http://www.genecards.org/) and ZFIN (http://zfin.org/). The distribution of teleost-specific genes (teleost^+^ as compared with tetrapods) along channel catfish chromosomes was visualized within 1-Mb bins using Circos^62^.

Similarly, the zebrafish–elephant shark (*Callorhinchus milii*) and zebrafish–lamprey (*Petromyzon marinus*) orthologue sets were generated using InParanoid software to produce the ‘cartilaginous fish orthologue core set'. The genes present in teleosts but absent in the shark and/or lamprey were identified first by comparison of the ‘teleost ortholog core set' with the ‘cartilaginous fish orthologue core set'. This set was then further curated manually using BLAST searches against the genome assemblies of sea lamprey, elephant shark, whale shark, *Rhincodon typus* and transcriptome assemblies of small-spotted catshark (*Scyliorhinus canicula*) and little skate (*Leucoraja erinacea*; retrieved from Skatebase http://skatebase.org/) with a maximal e-value of 1e−5. Teleost genes that matched any cartilaginous fish and/or lamprey sequences were excluded for further analysis. The distribution of teleost-specific genes (teleost^+^ as compared with cartilaginous fish and/or lamprey) along the channel catfish chromosomes was visualized within 1 Mb bins using Circos^62^.

The maximum-likelihood phylogeny of species under analysis was constructed in MEGA5 software^67^, using the Jones–Taylor–Thornton (JTT) substitution model, nearest-neighbor-Interchange maximum-likelihood heuristic method using default parameters. Multiple sequence alignment of concatenated protein sequences of 1,693 genes with a 1:1 ratio of orthologues in all these species were performed using ClustalW implemented in MEGA with default parameters, followed by complete deletion of gaps.

### Status of known scale development genes in catfish

The zebrafish genes encoding five genes involved in scale development (*EDA, EDAR, FGFR1A, LEF1* and *TCF7*)^22^,^23^,^24^, were used as queries against the channel catfish skin RNA-seq data using TBLASTN with maximal e-value of 1e−5. The catfish transcript sequences were then queried against the genome assembly, and open reading frames were compared with those of zebrafish or fugu using ClustalW2 (http://www.ebi.ac.uk/Tools/msa/clustalw2/).

### Scale regeneration experiments

All procedures involving the handling and treatment of fish used during this study were approved by the Auburn University Institutional Animal Care and Use Committee (AU-IACUC) before initiation of the project. Experimental common carp, *Cyprinus carpio*, were anaesthetized by immersion in MS-222 solution (100 p.p.m., pH 7.0). To determine differentially expressed genes during carp scale regeneration, scales were removed from a 3 cm × 3 cm area on the left side of each fish. Fish were killed at various time points and skin samples were collected from the descaled area of nine fish for each time point of 0 h, 12 h, 24 h, 3 days, 5 days, 7 days, 14 days, and 21 days. Daily observations were made during the regeneration experiments. Regenerated scales began to emerge ∼5 days after scale removal and were macroscopically visible at 12–14 days after scale removal. Skin tissues were frozen with dry ice and stored at −80 °C until isolation of RNA. A total of 72 carp were used in the experiments, and nine random individuals were sampled at each of the eight time points. Total RNA was isolated using the RNeasy Plus Universal Kit (Qiagen Inc., Valencia, CA). RNA-Seq analysis, including production of a *de novo* transcriptome assembly, was conducted as previously described^24^,^26^,^28^.

### Differentially expressed genes during scale regeneration

To identify differentially expressed genes during carp scale regeneration, the trimmed reads from each time point were mapped to the *de novo* assembly using CLC Genomics Workbench. Parameters for mapping the reads to reference transcriptome assembly were a minimum alignment of 90% of the transcript length and a maximum of two base mismatches. Total mapped reads were counted for each transcript and then normalized to produce ‘reads per kilobase of exon model per million mapped reads' (RPKM). Differentially expressed genes required a minimum twofold change in expression with a FDR-corrected *P* value <0.05.

### Interspecific comparative transcriptome analysis

Total RNA was isolated from the skin tissues of channel catfish and common carp using the RNeasy Plus Universal Kit (Qiagen, CA) according to the manufacturer's instructions. Raw sequencing reads were filtered for base quality ≥15 and read length ≥30 bp. The *de novo* assembly was produced using Trinity (version r2012-06-08)^68^. The assembled contigs for each species were used as queries against the NCBI Non-Redundant protein database using BLASTX with maximum e-value of 1e−5, and only the best match was annotated for each contig.

Interspecific comparative skin transcriptome analyses between common carp (scaled) and channel catfish (scaleless) were conducted using TBLASTX with a E-value cutoff of 1e−5 with the following steps: (1) The carp skin transcriptome and the channel catfish skin transcriptome were *de novo* assembled, separately; (2) The carp skin transcriptome was annotated by BLASTX analysis against the non-redundant database; (3) A list of unique protein-coding transcripts from the common carp skin transcriptome were compiled and queried against the channel catfish skin transcriptome; (4) For carp contigs with no matches to the channel catfish transcriptome assembly but with matches to the non-redundant database, the sequences of each best match were retrieved from the non-redundant database and used to query the channel catfish skin transcriptome assembly. Those carp transcripts that remained unidentified were annotated as genes that were expressed in the carp skin but not expressed in the channel catfish skin.

### Phylogenetic analysis of SCPP genes

A phylogenetic tree was constructed from multiple sequence alignments of amino-acid sequences using the maximum-likelihood method in MEGA5 (ref. 67). Data were analysed using a JTT model, and gaps were removed by partial deletion. The topological stability was evaluated by 1,000 bootstrapping iterations. Sequences included in the analysis were retrieved from NCBI or from the channel catfish genome assembly.

### Expression analysis of SCPP genes using RT–PCR

Reverse-transcriptase PCR (RT–PCR) was used to study the messenger RNA expression of SCPP genes in zebrafish. To study the normal expression of these genes in healthy fish, skin, scale, bone, tooth, dorsal fin, ventral fin and caudal fin were pooled from eight individual fish. Three such pools were used in the present study. The tissues were snap-frozen in liquid nitrogen and immediately subjected to RNA extraction using RNeasy Plus Universal Kit (Qiagen, CA) following the manufacturer's protocol. Total RNA was quantified by ultraviolet-spectrophotometry and an aliquot (1 μg) of RNA was treated with 1 unit of RNase-free DNase (Qiagen) before reverse transcription. A uniform quantity of DNA-free RNA was reverse-transcribed using iScript cDNA Synthesis Kit (Bio-Rad, USA) following manufacturer's protocol. Touchdown PCR was performed using cDNA to analyse the expression levels of SCPP genes. The 10 μl PCR reaction mixture contained 1.0 μl 10 × buffer 0.5 μl of 50 mM MgCl2, 1.0 μl of 10 mM dNTPs, 0.25 μl (0.5 U) Platinum Taq polymerase, 10 pmol of each primer, 1 μl cDNA and 4.25 μl PCR-grade water. Gene-specific primers and reference gene-specific primers for ribosomal protein L13a (RPL13a)^69^ were used separately in the PCR amplification (Supplementary Table 12). Amplification was performed on a Bio-Rad PCR system with the PCR conditions as follows: (1) 94 °C for 5 min; (2) 6 cycles of 94 °C for 30 s, 56 °C for 30 s and 72 °C for 1 min; (3) 9 cycles of 94 °C for 30 s, 54 °C for 30 s and 72 °C for 1 min ; (4) 3 cycles of 94 °C for 30 s, 52 °C for 30 s and 72 °C for 1 min; (5) 17 cycles of 94 °C for 30 s, 48 °C for 30 s and 72 °C for 1 min; and (6) 72 °C for 10 min. The PCR products were resolved on a 1.2% agarose gel.

### Genome sequencing and analysis of the armored catfishes

Genomic DNA was isolated from blood from one individual each from two species of armored catfish, the common pleco and a southern striped Raphael. Small insert (400 bp) libraries were produced for each fish using standard protocols and sequenced on an Illumina HiSeq 2000 instrument to produce 100-bp paired-end reads. The *de novo* assembly was performed using ABySS (version 1.3.7)^60^ using multiple k-mers ranging from 31 to 96. Only contigs longer than 200 bp were included in the final assembly. Genome annotation was conducted using FGENESH program from MOLQUEST (version 2.4) package. The predicted amino-acid sequences were annotated by homology search against the NCBI Non-Redundant protein sequence database using the BLASTP program with a maximal e-value of 1e−5.

### Comparative genome analysis of scaled and scaleless catfish

We conducted comparative genome analysis between the armored catfish and channel catfish to identify candidate genes for scale development. Annotated genes from the two armored catfish were pooled and compared with the channel catfish genes using BLASTN with a maximal e-value of 1e−5. The identified genes were further refined by BLASTN searched against additional channel catfish genomic resources including whole-genome resequencing data^22^, RNA-Seq data sets^24^,^26^,^28^, ESTs^25^ and BAC end sequences^21^. We recognized the potential that the gene predictions for the channel catfish and armored catfish data sets could be incomplete and resulting in unmatched orthology. To resolve this issue, genes from the GenBank non-redundant database corresponding to incomplete armored catfish genes were retrieved and used as queries to BLAST against all channel catfish genomic resources. Only the genes without a BLAST match were determined as the armored catfish ‘specific' genes that were present in the armored catfish but not present in the channel catfish genome. Gene ontology analysis in combination with knowledge based on literature searches was carried out to functionally annotate these genes and identify genes potentially involved in scale development. Accordingly, we conducted a detailed analysis of the SCPP genes that have been previously investigated for their potential involvement in scale development.

### Transcriptome analysis of common pleco skin

Total RNA was isolated from the skin tissues of the common pleco using the RNeasy Plus Universal Kit (Qiagen, CA) according to the manufacturer's instructions. RNA-Seq was conducted as described above. The *de novo* assembly was produced using Trinity (version r2012-06-08)^68^; the assembled transcripts were annotated by homology searching against the NCBI Non-Redundant protein database using BLASTX with a cutoff e-value of 1e−5. Comparative subtraction of the channel catfish and common pleco transcriptomes was conducted as detailed above.

### Data availability

All sequence data that support the findings of this study have been deposited in GenBank with the following accession numbers: LBML01000000 for whole-genome sequence assembly under BioProject accession PRJNA281269; SRX1002658 for sequences of the 400 bp libraries, SRX1004615 for those from the 3 kb libraries, SRX1004616 for those from the 8 kb libraries and SRX100461 for those from the 34 kb fosmid libraries; SRX1201393 for RNA-Seq data set for common pleco skin transcriptome, SRX1201398 for carp skin transcriptome, and SRX1003286 and SRX1004613 for channel catfish skin transcriptome; SRX1201143 for common pleco genomic sequences, and SRX1201391 for short reads of southern striped Raphael genomic sequences.

## Additional information

**Accession codes:** Sequence data have been submitted to the BioProject database under accession PRJNA281269. This whole-genome assembly has been deposited at DDBJ/EMBL/GenBank under the accession LBML00000000. The version described in this paper is version LBML01000000. The channel catfish genomic sequences produced were submitted under this BioProject with accession numbers of SRX1002658 for sequences of the 400 bp libraries, SRX1004615 for those from the 3 kb libraries, SRX1004616 for those from the 8 kb libraries, SRX100461 for those from the 34 kb fosmid libraries. RNA-Seq data sets were submitted under this BioProject under the accession numbers of SRX1201393 for Pleco skin transcriptome, SRX1201398 for carp skin transcriptome, and SRX1003286 and SRX1004613 for channel catfish skin transcriptome. The short reads for common pleco and southern striped Raphael draft genome sequence assembly has been deposited at the NCBI Sequence Read Archive (SRA) under accession number SRX1201143 and SRX1201391, respectively.

**How to cite this article:** Liu, Z. *et al*. The channel catfish genome sequence provides insights into the evolution of scale formation in teleosts. *Nat. Commun.* 7:11757 doi: 10.1038/ncomms11757 (2016).

## Supplementary Material

Supplementary Information Supplementary figures 1-11, Supplementary Tables 1-12

Supplementary Data 1 A list of 111 genes included in the most highly variable genomic regions.

Supplementary Data 2 A list of 3,688 duplicated gene sets in channel catfish.

Supplementary Data 3 GO enrichment analysis of recently duplicated genes (Ks<1.0).

Supplementary Data 4 A list of 297 genes commonly shared by all sequenced teleost fish species but absent from tetrapod species.

Supplementary Data 5 A list of 280 genes commonly shared by all sequenced teleost fish species but absent from cartilaginous fish and jawless species.

Supplementary Data 6 A list of 100 genes commonly shared by all sequenced teleost fish species but absent from tetrapods, cartilaginous fish and jawless species.

Supplementary Data 7 A list of 836 genes that were expressed in the skin of common carp, but not in the skin of channel catfish.

Supplementary Data 8 A list of 1,173 genes that were differentially expressed during carp scale regeneration.

Supplementary Data 9 A list of 169 genes that were present in pleco or doras, but absent from channel catfish.

Supplementary Data 10 A list of 704 genes that are expressed in the skin of scaled pleco but not in scaleless channel catfish.

## Figures and Tables

**Table 1:** Summary statistics of the channel catfish reference genome assembly.

Assembly statistics	Contig number	Contig length (bp)	Scaffold number	Scaffold length (bp)
N50	2,839	77,200	31	7,726,806
N90	10,984	16,103	185	498,561
N95	14,209	8,426	314	190,570
N98	18,709	2,632	594	28,074
N99	23,055	1,291	1,927	1,827
Max	−	607,423	−	22,613,484
Average	−	22,301	−	78,523
Total	34,615	771,933,303	9,974	783,193,925
Anchored to chromosomes	−	−	634	758,102,267 (96.8%)
