# Supplementary material for: The channel catfish genome sequence provides insights into the evolution of scale formation in teleosts
Source: Nat Commun. 2016 Jun 2;7:11757. doi: 10.1038/ncomms11757 (PMC4895719; doi:10.1038/ncomms11757)
Supplement: Supplementary Information — Supplementary figures 1-11, Supplementary Tables 1-12 [file ncomms11757-s1.pdf]

Supplementary Information

Supplementary Figures

Supplementary Figure 1. Estimation of genome size from analysis of k-mers from raw sequence of the Illumina short insert library.
Genome size was calculated as total trusted sequence divided by the multiplicity at the mode. Genome size estimate ranged from 1.008-1.013 Gb. MaSuRCA automatically utilized a kmer length of 31 which produced a genome size estimate of 1.008 Gb based on 88.7 Gb of trusted sequence. In comparison, the genome size estimated from 5.1 Gb of trusted 31-mer sequence from the Illumina-corrected PacBio sequence was 1.021 Gb.

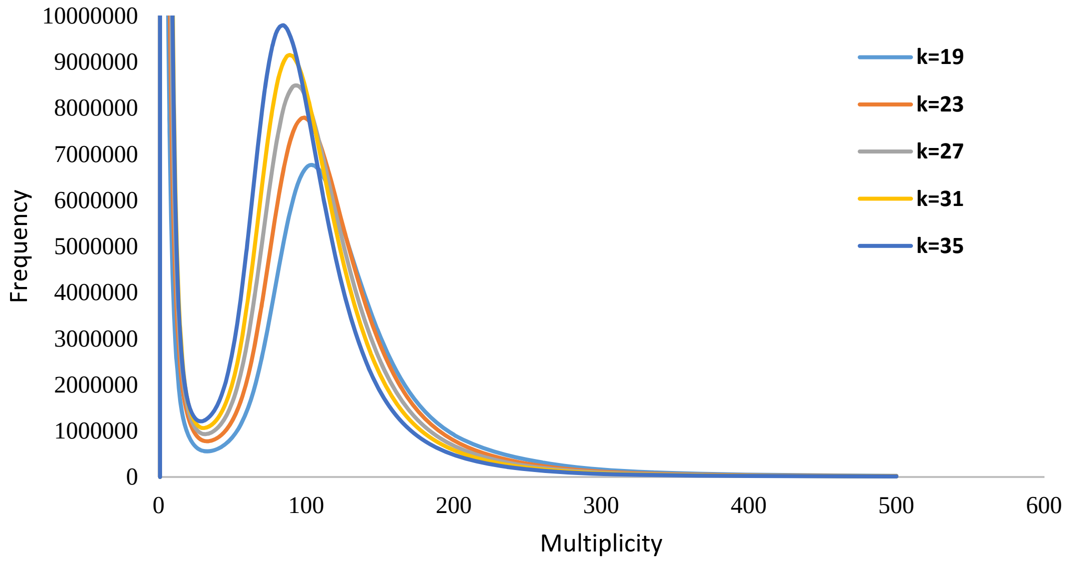

Supplementary Figure 2a. Concordance of assembled scaffolds with genetic map for chromosomes 1-6. Scaffolds spanning > 3 cM with minimal overlap are included in plots.

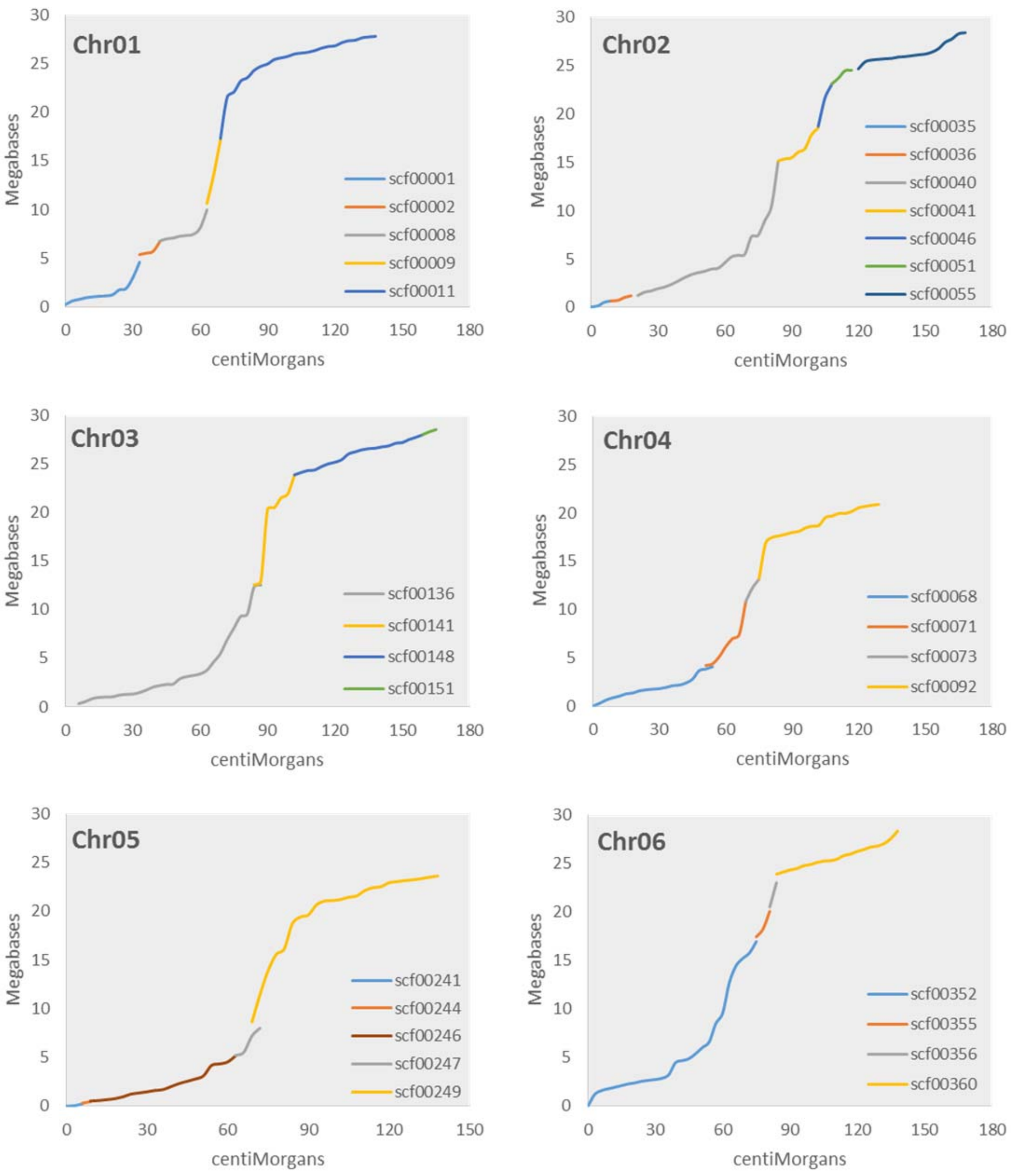

Supplementary Figure 2b. Concordance of assembled scaffolds with genetic map for chromosomes 7-12. Scaffolds spanning > 3 cM with minimal overlap are included in plots.

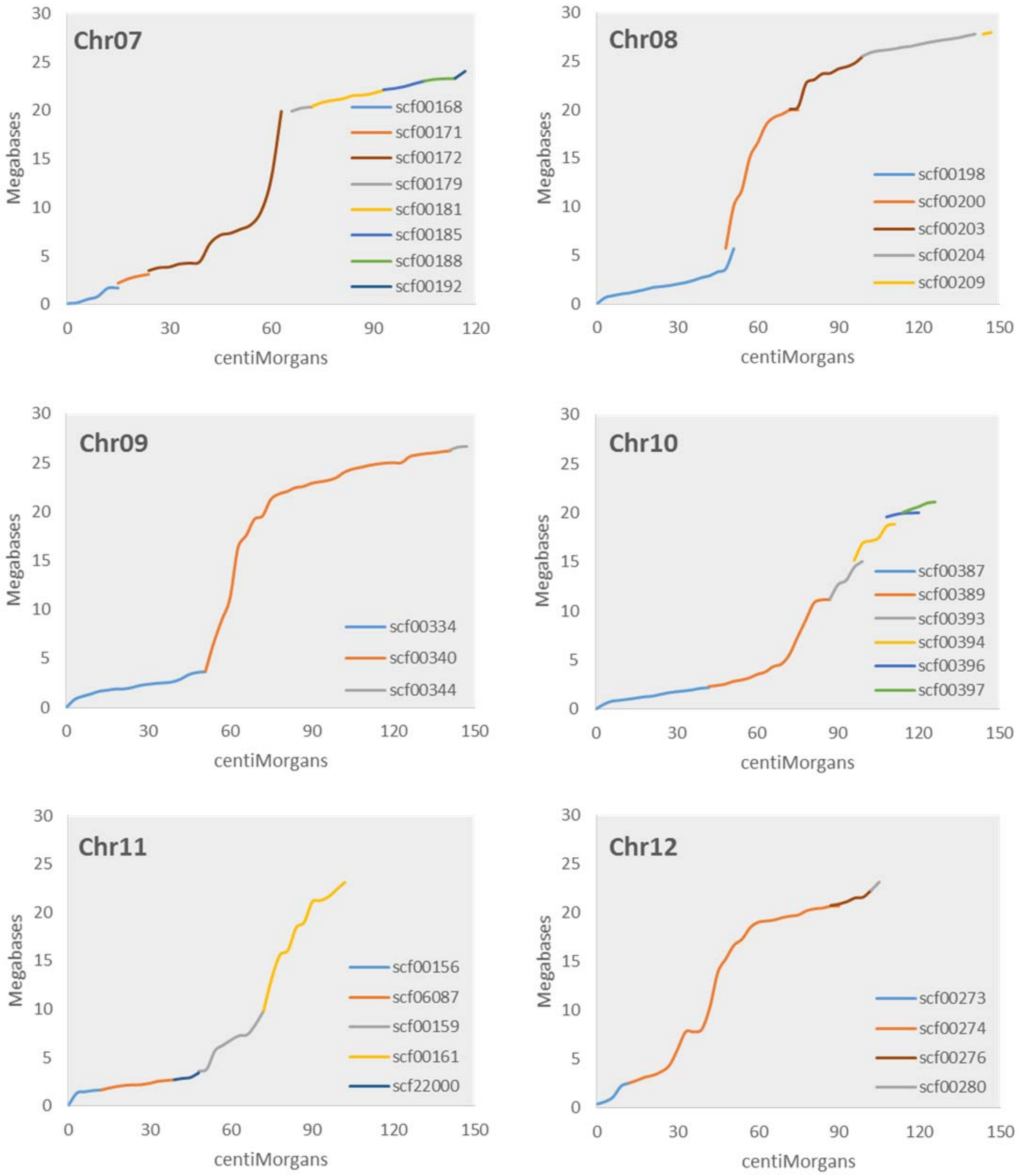

Supplementary Figure 2c. Concordance of assembled scaffolds with genetic map for chromosomes 13-18. Scaffolds spanning > 3 cM with minimal overlap are included in plots.

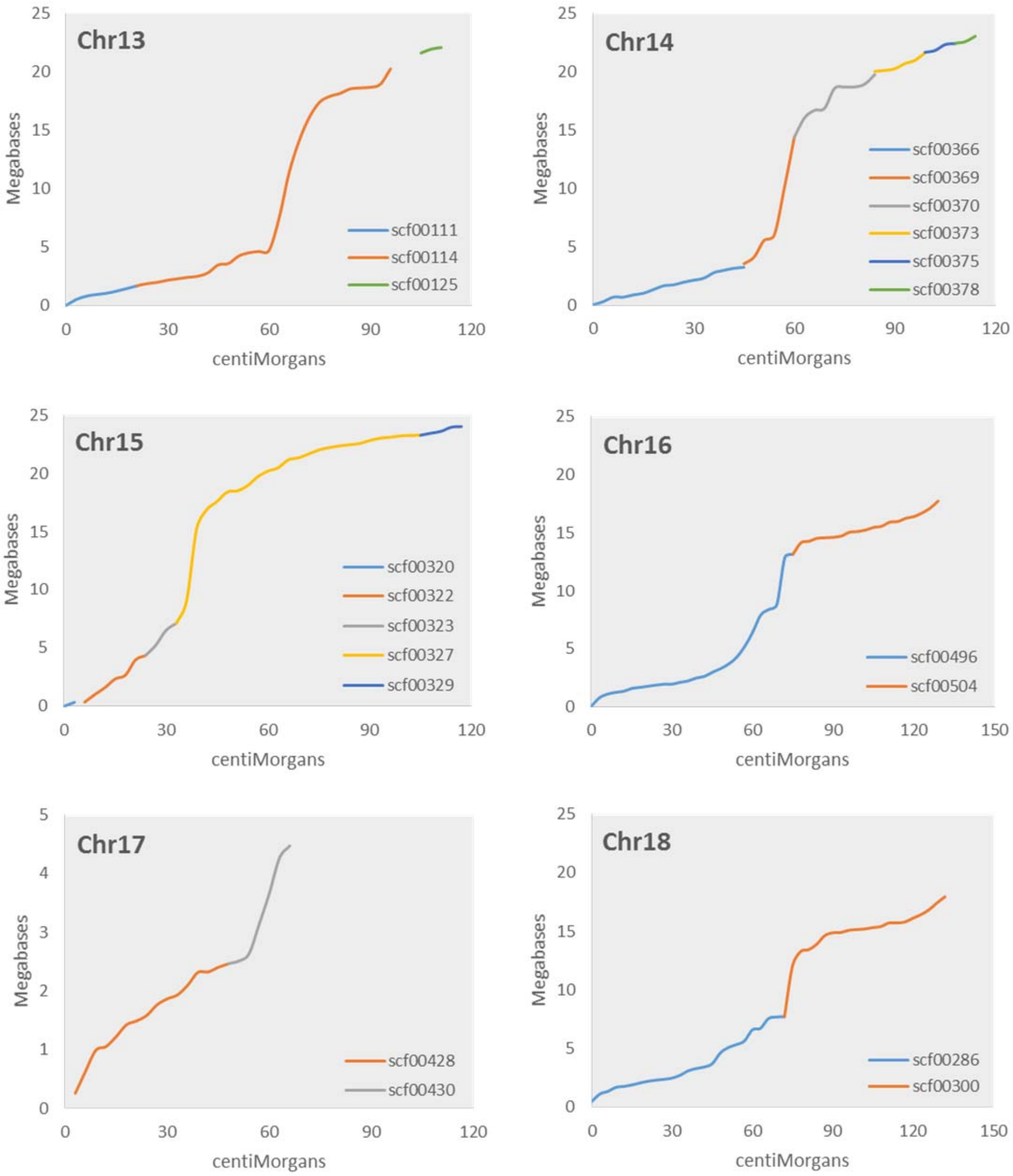

Supplementary Figure 2d. Concordance of assembled scaffolds with genetic map for chromosomes 19-24. Scaffolds spanning > 3 cM with minimal overlap are included in plots.

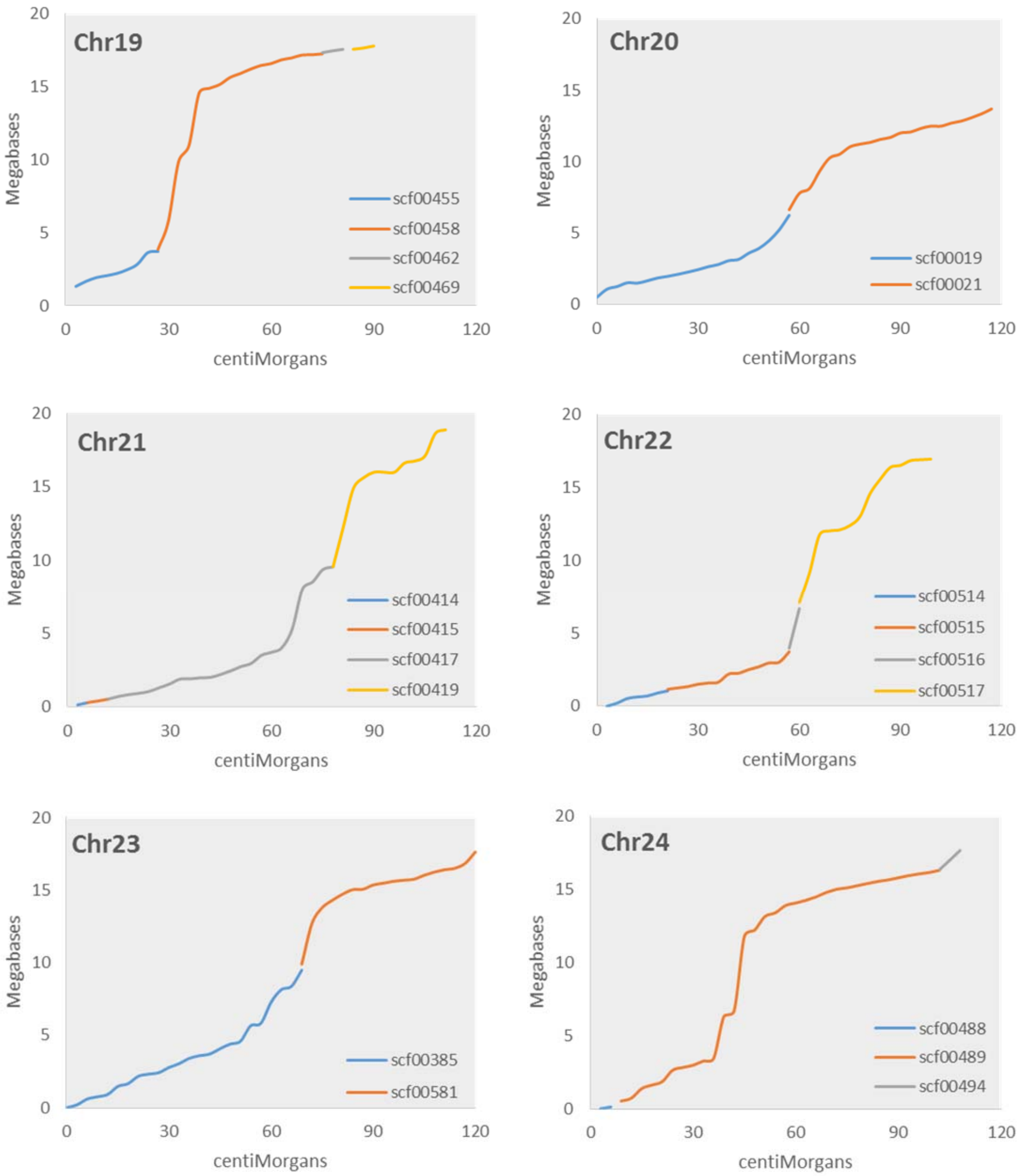

Supplementary Figure 2e. Concordance of assembled scaffolds with genetic map for chromosomes 25-29. Scaffolds spanning > 3 cM with minimal overlap are included in plots.

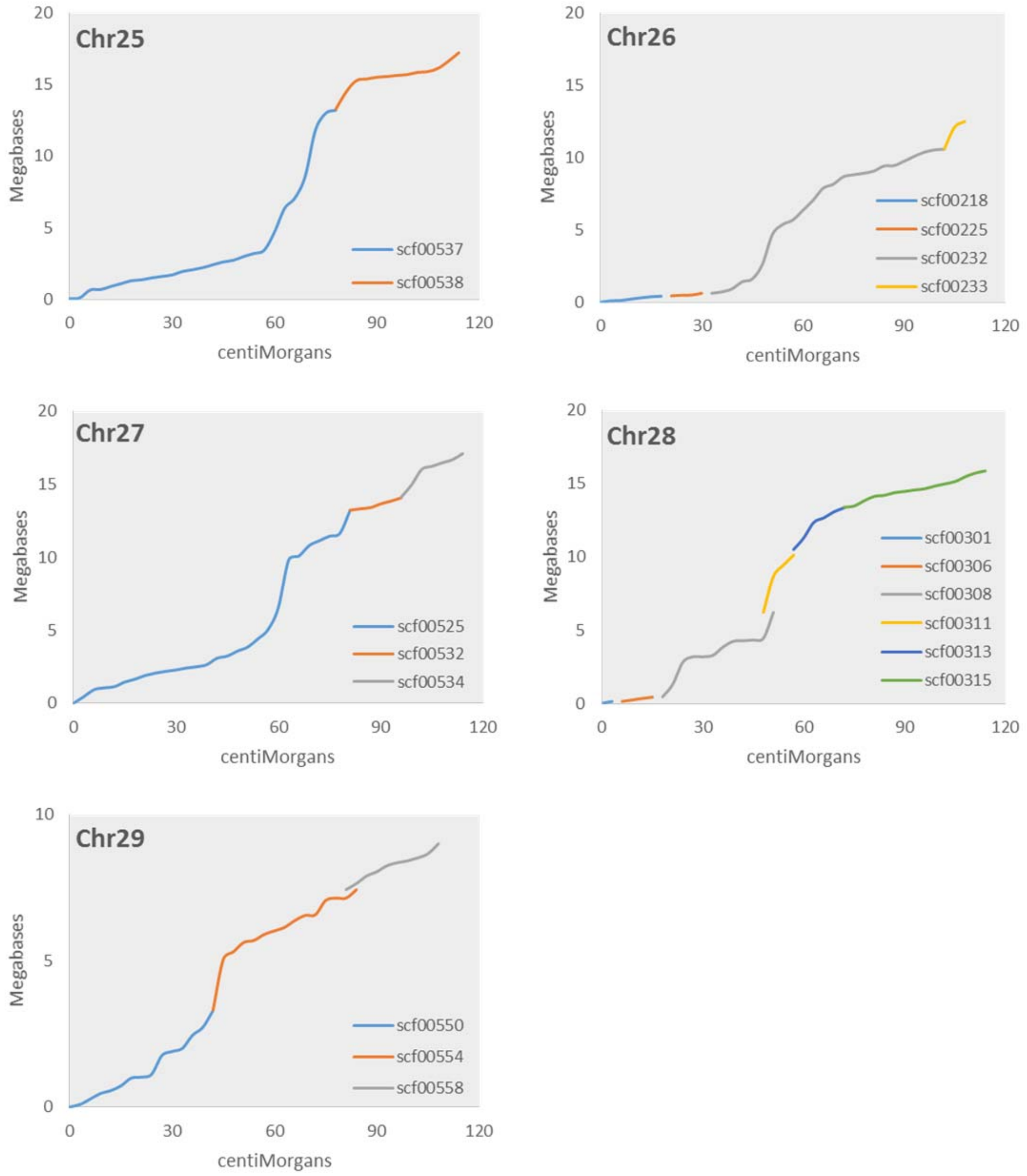

Supplementary Figure 3. Alignments of 167X genome coverage of reads from re-sequencing of 150 individuals.
Note that the reference genome is fully covered by the assembled contigs of the re-sequencing reads.

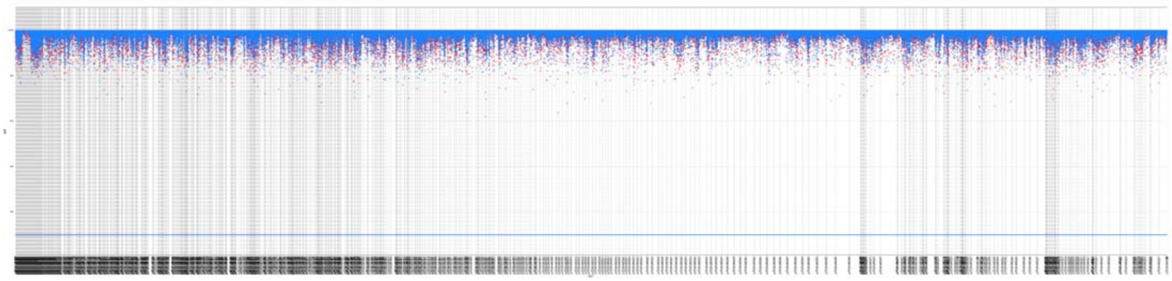

Supplementary Figure 4. Analysis pipeline for the coding potential of the channel catfish genome.

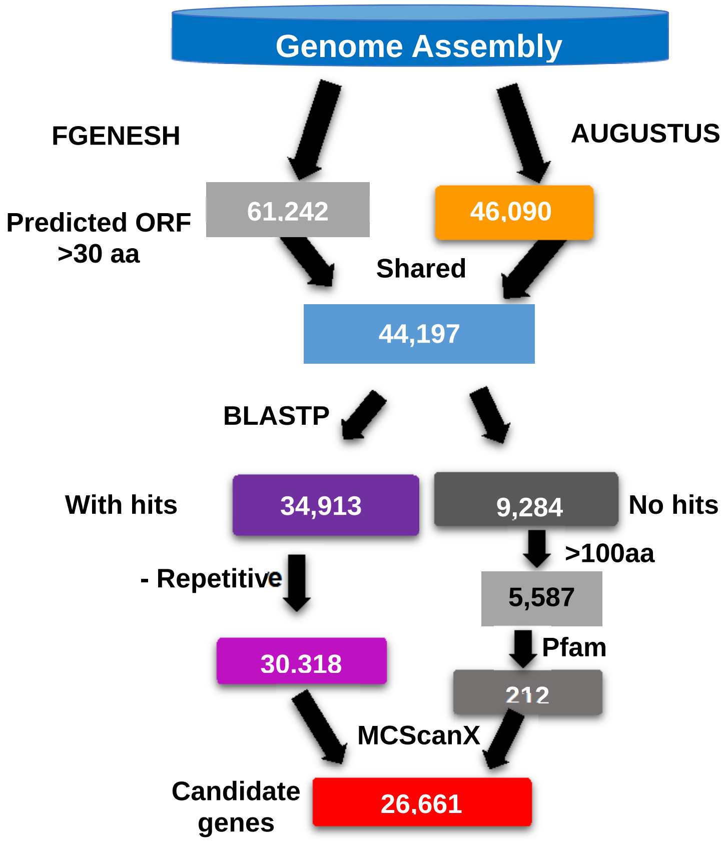

Supplementary Figure 5. Chromosome (chr) length in Mb (A), distribution of genes (B), single nucleotide variations (C), and teleost-specific genes when compared with tetrapods (D, outward red lines) and when compared with cartilaginous fish (D, inward blue lines), across the channel catfish genome. For B, C, and D, the x-axis corresponds to chromosomal locations as depicted in A, while the Y-axis represents number of genes in B, number of SNPs in C, and teleost-specific genes in D.

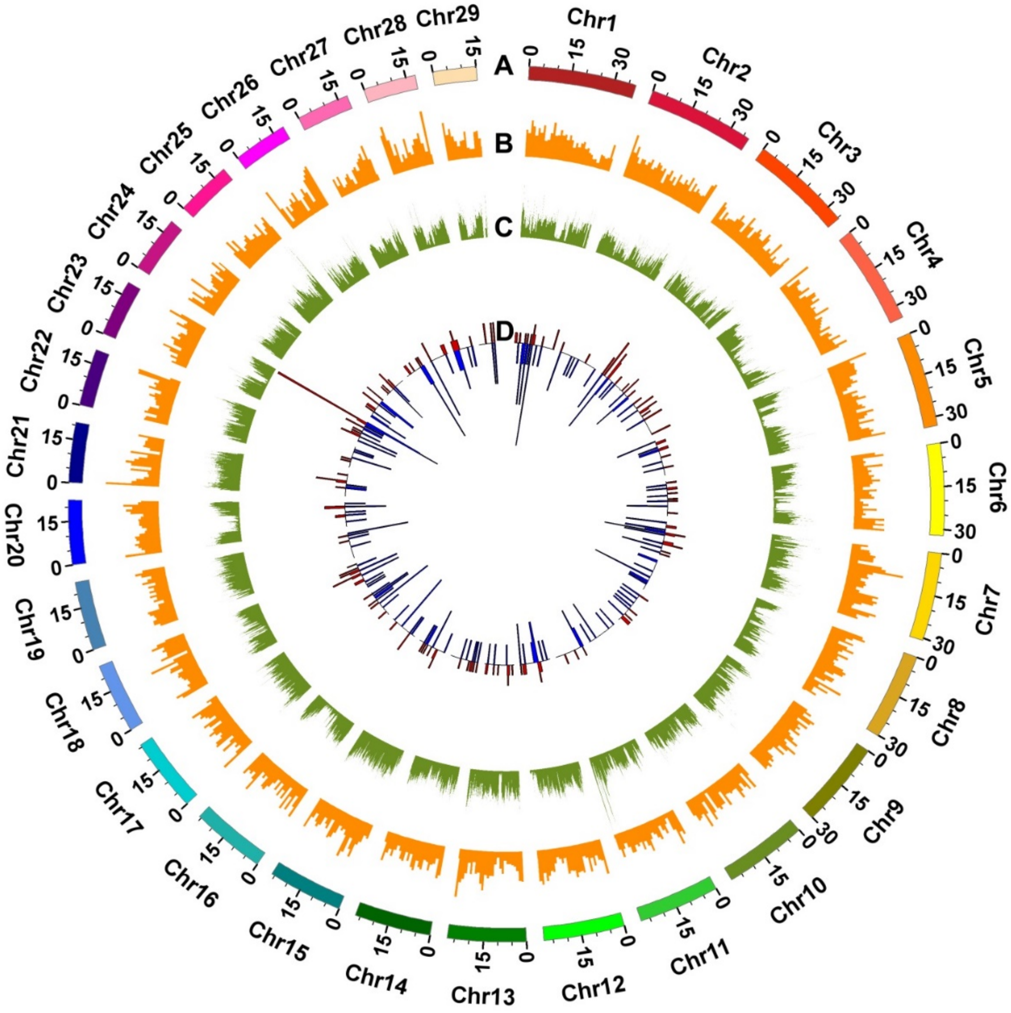

Supplementary Figure 6. Analysis of orthologies of channel catfish and zebrafish genes.

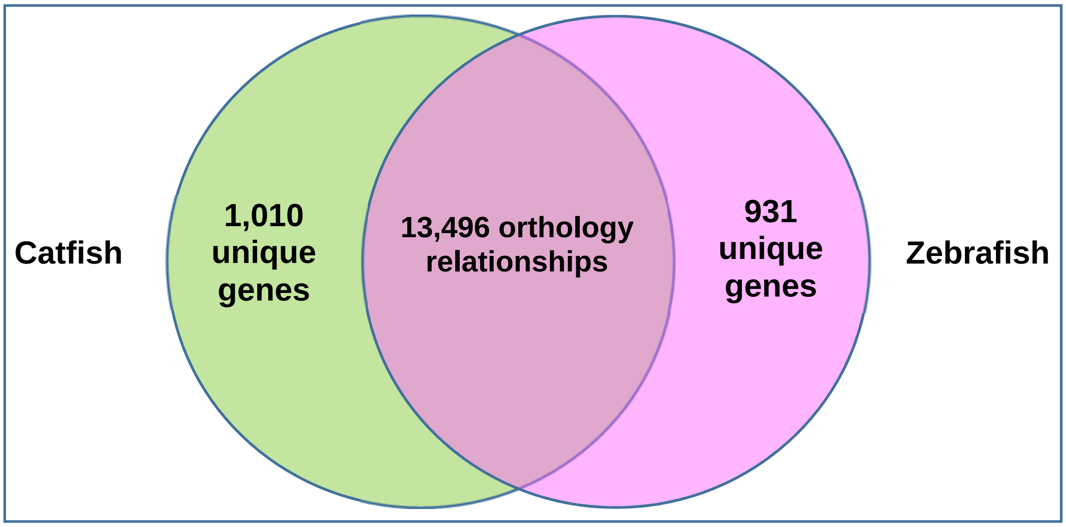

Supplementary Figure 7. Sequence alignment of Eda of channel catfish (channel) with that of zebrafish.

```
channel         ----MGSVTESPEESAS-----KPAVKCVCQSRCGGGGGGSWKAFAGLFALSLALHLVTL 51
zebrafish       MLIDLHAAMEPKREPAKLEERERTQAACECQSQCN-----NCKIFLSLFILSLSLHLVTL 55
                    : :. *. .*.*.     :. . * ***:*.     . * * .** ***:******
channel         ACYVELRSEVEREIRIQK----SGSG-----AEAPPVPARGGGGGGEVHEQIRVEREAE- 101
zebrafish       FCYLDLRSELKREISQKNKDEVSSTGPVPHYEATEPVLQSPDTDHPTIGDQSRWRDEHTR 115
                 **::****::***  ::    *.:*       : **    . .   : :* * . *
channel         --EKIILRSKRSESPGNGKRKGERKKGKKGPPGAPGPPGPPGPQGPPGIPGIPGIPGSNA 159
zebrafish       GLERVIHRTKRSET--NGKKKGERKKGKKGPPGAPGPPGPPGPQGPPGIPGIPGIPGSNA 173
                  *::* *:****:  ***:****************************************
channel         MGP---PGPPGPAGPPGPQGPPGAQGTPGGGERSRFRDTQLAVVHLQGQETTIQVKEDLS 216
zebrafish       MGPSGPPGPPGPQGPPGPQGPPGPQG-PSGGEKGKHREAQPAVVHLQGQETTIQVKEDLS 232
                ***   ****** **********.** *.***:.:.*::* *******************
channel         EGVLKNWKTIAMHQRVFKMHSRSGELEVLQDGMYFIYSQVEVYFLNFTDIASYEVMIDKD 276
zebrafish       EGVLKNWRMISIHQRVFKMHSRSGELEVLLDGTYFIYSQVEVYYLNFTDIASYEVMVDKT 292
                *******: *::***************** ** **********:************:**
channel         PFLRCTRSIETGQRKFNTCYTAGVCWLRARQRISIRMVYDDTSISMTNHTTFLGSIRLGE 336
zebrafish       PFLRCTRSIETGQRKFNTCYTAGVCLLRARQRISIRMVYEDTSISMSNHTTFLGSIRLGD 352
                ************************* *************:******:************:
channel         APPSGHS 343
zebrafish       APSAGHT 359
                **.:**:
```

Supplementary Figure 8. Sequence alignment of Edar of channel catfish (channel) with that of zebrafish.

```
channel         MARKIPRVESCLLSFLLASSVYPLRAEYSSCGENEFYNHSSSSCRPCPQCQPGQEPYMNC 60
zebrafish       MGHKGGRTPSFLLHFLLVSSMSTVSAEYSSCGENEFYNHTTSSCQPCPQCQAGQEPYMNC 60
                *.:*  *. * ** ***.**: .: **************::***:******.********

channel         GYGVKDEDYDCVSCPPGKFSRGKYEICRRHKDCNALYKATVLTPGTRESDAECGACLPGY 120
zebrafish       GYGTKDDDYSCVSCPAGKFSKGKYEICRRHKDCDALYRATVLTPGTSDSDAECGHCLPGY 120
                ***.**:**.*****.****:************:***:******** :****** *****

channel         FILENRLRNIYSMMCHSCQNAPRNTKECMRTTSTSTKHVPGIPSTSTTTFPHAQKDPTGQ 180
zebrafish       YIQENRPQNIYGMVCHSCQNAPRNIKECMRSTPPASGRAPSVSSSTTIFPQPEKDPTGQ 180
                :* *** :***.*:********** *****:*..:: :.*.:.*:*** **:.:******

channel         GHLATALIIAMSTIFIMAIAIVMIIMFYILKAKTSGQACCSGQIVKTIEAPMNKQEEKKE 240
zebrafish       GHLATALIIAMSTIFIMAIAIVMIIMFYILKSKPSGPVCCSGQLIKAVEAQTNMQEEKKE 240
                *******************************:*.** .*****::*::**  * ******

channel         AQENAVIFTEKDEF---KPPPTKAVKSENDASSENEQLLSRSIDSDEEAAQEKQGATDLC 297
zebrafish       AQENVVIFQEKDEFDKLKPSSPKTAKSENDASSENEQLLSRSIDSDEEAAQDKQGAADLC 300
                ****.*** *****   **...*:.**************************:****:***

channel         LLSLVHLTRDKTCSNNTVSTTVNNNNNNCSNNSRTMGIHSRRKKILELYAKACGVAEGVS 357
zebrafish       LLSLVHLTRDKXCX----TNTINNNNNHCS---RATGIHSRRKKILDLYTKACSVAEGLS 353
                *********** *     :.*:*****:**   *: **********:**:***.****:*

channel         PVELPFDCLERTSRMLSTTYSAEKALVRTWRHLAESFGLKRDEIGGMTDGMQLFDRVSTA 417
zebrafish       PTELPFDCLERSSXMLSATYSTDKAVVKTWRHLAESFGLKRDEIGGMTDGMQLFDRISTA 413
                *.*********:* ***:***::**:*:**************************:***

channel         GYSIPDLLARLVQIERLDVVEALCFDILGAPPSGQHLTISSRCASV 463
zebrafish       GYSIPDLLARLLQIERLDAVETLCCDILSGTQNCTNPPLSSRCASV 459
                ***********:******.**:** ***... .  : .:*******
```

Supplementary Figure 9. Sequence alignment of fgfr1a of channel catfish (channel) with that of zebrafish.

```
channel         VAFVHRHCPSSPSTEQLESCVYGELLQATSSVVEEHDEEHNEGHAAAVLGSSSPASAGPQ 60
zebrafish       ------------------------------------------------------------

channel         QAGQPRARTSGYGTVSPVSPSGRADMVRSRPCLRQDERIWNGLARVSVRTSGYGTVSPVS 120
zebrafish       -------------------------MIMK-----------TTLLLISVLLT--------- 15
                                         *: .           . *  :**  :
channel         PSGRADMERSRPCLRQDEWIWNCLLRVALLETAVYTLYEGDKLELKCRSKEEPQEVSWTK 180
zebrafish       ---QALQSQGRPAIQDE----------APAEPTSYTLDSGEKLELSCKAKEDTQKVTWTK 62
                   :*  .:.**.::::          *  *.: *** .*:****.*::**:.*:*:***
channel         DQVLLADGEHTHLRNGQLEIEGVELADSGLYGCFARGPAGNHTEYFNVNVTYALASSEDD 240
zebrafish       DLVPLVDGEHTRLRNDQMEIEKVEPADSGLYACFAQGLNSNHTEYFNISVT-------DE 115
                * * *.*****:***.*:*** ** ******.***:*  .*******:.**       *:
channel         DDDDESSSEETKQSSSQKLLSNSQELSPTAPEWAQPDKMEKKLHAVPASRTVRFRCPSIG 300
zebrafish       EDEVDSSSEEAK-------LSNDQNL-PMAPVWAQPDKMEKKLHAVPASKTVKFRCQANG 167
                :*: :*****:*       ***.*:* * ** ****************:**:*** : *
channel         NPTPTLKWLKNGKEFKKDQRIGGFKLRESLWAIIMDSVVPSDKGNYTCVVENKYGSINHT 360
zebrafish       NPTPTLKWLKNGKEFKRDQRIGGFKVREHMWTIIMESVVPSDRGNYTCLVENRHGSINHT 227
                ****************:********:** :*:***:******:*****:***::******
channel         YQLDVVERSPHRPILQAGLPANRTAVVGSDVEFECKVFSDPQPHIQWLKHIEVNGSKVGE 420
zebrafish       YQLDVVERSPHRPILQAGLPANRTAVVGSDVEFECKVFSDPQPHIQWLKHIEVNGSRYGP 287
                ********************************************************: *
channel         DGLPYVKVLKHSGVNSSDTQVLTLHNVTEEESGEYICKVSNYIGQANQSAWLTVTKYAST 480
zebrafish       DGLPYVRALK-------------------------------------------------- 297
                ******:.**
channel         ETAGVNTTDKEMEVLQLKNVSLEDAGVYTCLAGNSIGHSHHSAWLTVYEALPPTPLPNQT 540
zebrafish       -TAGVNTTDKEMEVLQIRNVSLEDAGEYTCLAGNSIGHSHHSAWLTVYKAVPPTQLPNQT 356
                 ***************::******** *********************:*:*** *****
channel         YLEVLIYCVGFFLICVMVVIAVLVKMHSSAKKSDFNSQMAVHKLAKSIPLRRQV--SVDS 598
zebrafish       YLEVLIYCVGFFLICVMVGTAVLAKMHSSAKKSDFNSQLAVHKLAKSIPLRRQVTVSVDS 416
                ******************  ***.**************:***************  ****
channel         SSSMHSGVMLVRPSRLSSSGSPMLSGVSEYELPQDPRWELPRDKLVLGKPLGEGCFGQVV 658
zebrafish       SSSMHSGGMLVRPSRLSSSGSPMLSGVSEYELPQDPRWEVQRDRLVLGKPLGEGCFGQVM 476
                ******* *******************************: **:***************:
channel         MGEVLGMDKDKPNRVTKVAVKMLKSDATEKDLSDLISEMEMMKIIGKHKNIINLLGACTQ 718
zebrafish       MAEAMGMDKEKPNRITKVAVKMLKSDATEKDLSDLISEMEMMKIIGKHKNIINLLGACTQ 536
                *.*.:****:****:*********************************************
channel         DGPLYVIVEYASKGNLREYLRARRPPGMEYCYNPDQVPVETVSVKDLVSCAYQVARGMEY 778
zebrafish       DGPLYVIVEFAAKGNLREYLRVRRPPGMEYCYNPDQVPVENMSIKDLVSCAYQVARGMEY 596
                *********:*:*********.******************.:*:****************
channel         LASKKCIHRDLAARNVLVTEDNVMKIADFGLARDIHHIDYYKKTTNGRLPVKWMAPEALF 838
zebrafish       LASKKCIHRDLAARNVLVTEDNVMKIADFGLARDIHHIDYYKKTTNGRLPVKWMAPEALF 656
                ************************************************************
channel         DRIYTHQSDVWSFGVLLWEIFTLGGSPYPGVPVEELFKLLKEGHRMDRPSTCTHELYMMM 898
zebrafish       DRIYTHQSDVWSFGVLLWEIFTLGGSPYPGVPVEELFKLLKEGHRMDRPSTCTHELYMMM 716
                ************************************************************
channel         RDCWHAAPSQRPTFKQLVEDLDRTLSMTSNQEYLDLSVSLDQYSPSFPDTRSSTCSSGED 958
zebrafish       RDCWHAVPSQRPTFKQLVEDLDRTLSMTSNQEYLDLSVSLDQFSPNFPDTRSSTCSSGED 776
                ******.***********************************:**.**************
channel         SVFSHEPSADEPCLPKFPPNPNRVVSFKKR 988
zebrafish       SVFSHDAGADEPCLPKFPPHPNRGVAFKKR 806
                *****:..***********:*** *:****
```

Supplementary Figure 10. Sequence alignment of Lef1 of channel catfish (channel) with that of zebrafish.

```
channel         MPQLAGGGGGGDPELCATDEMIPFKDEGDPQKEQSFAEITNSEEEGDLAELKSSLVNESE 60
zebrafish       MPQLSGGGGGGDPELCATDEMIPFKDEGDPHKEQIFAEISHSEEEGDLAEIKSSLVNETE 60
                ****:*************************:*** ****::*********:*******:*

channel         TSPN-NSHDAARQSQITPDSYHEKHREHPDDGKLQDLYSKGHAYSGYPSYIMMTNMNNEP 119
zebrafish       ISPNSNSHDAARQSQITPDSYHEKHRDHPDDGKLQDLYSKGHPYPSYPGYIMMTNMNNEP 120
                 *** *********************:***************.*..**.***********

channel         YMNNGSLSPPMPRTSNKVPVVQPSHAVHPLTPLITYSDEHFAPGPHSGHHPQDSK----G 175
zebrafish       YMNNGSLSPPIPRTSNKVPVVQPSHAVHPLTPLITYSDEHFAPGPHSGHHPQDVNPKQAG 180
                **********:****************************************** :    *

channel         MPRHHPGPDIPNFYSLSPGGVGQLTPPLGWFPHHMVPGPPGPHATGIPHPAIVNPQVKHE 235
zebrafish       MPRHHPGPDIPNFYPLSPGGVGQMTPPLGWFSHHMVPGPPGPHATGIPHPAIVNPQVKQE 240
                **************.********:*******.**************************:*

channel         PPHETDLMHMKPQHEQRKEQEPKRPHIKKPLNAFMLYMKEMRANVVAECTLKESAAINQI 295
zebrafish       --HDTDLMHMKPQHEQRKEQEPKRPHIKKPLNAFMLYMKEMRANVVAECTLKESAAINQI 298
                  *:********************************************************

channel         LGRRWHALSREEQAKYYELARKERQLHMQLYPGWSARDNYGKKKKRKREKLQESASGTAQ 355
zebrafish       LGRRWHALSREEQAKYYELARKERQLHMQLYPGWSARDNYGKKKKRKREKIQEPASGTGQ 358
                **************************************************:**.****.*

channel         RMKTAYI 362
zebrafish       RMKTAYI 365
                *******
```

Supplementary Figure 11. Sequence alignment of TCF7 of channel catfish (channel) with that of zebrafish.

```
channel         KMPQLNSGGGDDLGANDEMIAFKDEGEQDEKIEENAFTEGDLADLKSSLVNESEISQNSH 60
zebrafish       -MPQLNGGGGDDLGANDEMIAFKDEGDHEEKIRESAFTESDLADLKSSLVSETEISQS-- 57
                 *****.*******************:.:***.*.****.**********.*:****.

channel         PPVVRTGHQEEQRVYEEKHREHLEHLNDVPKPQDAGMYKASTYSGYPFLMLPEHYHPNGQ 120
zebrafish       PAVIRRGQQDEQRIYSDKR----EHLDDVPKHHDGGMYKAP-YSGYPFLMLPEPYLPNGP 112
                *.*:* *:*:***:*.:*:    ***:**** :*.*****. *********** * ***

channel         VSPSANKVAVVQQPHGMHPLTSLLPYSNEHFNPSPSHLTSDMSQKPGGVGQITPSMGWQS 180
zebrafish       VSPPSNKVSVVQQ--GMHPLTPLLPY--EHFNPSPTHMPTDGGQKPG-------VHRHQT 161
                ***.:***:****  ******.****  *******:*:.:* .****           *:

channel         QPVYPLSSCGFRQPYSSSLASNSSYSRFSHSLIMGPSGMHPTGIPHPAIVPSSGRQEHDQ 240
zebrafish       QEIS-----GFYSLPQGQITP--SMNWFSHSLMLQ-SGMHPTGIPHPAIVPPSGKQEHDQ 213
                * :      ** .  ...::.  * . *****::  **************.**:*****

channel         FD----IKPHPESKREREPKKPVIKKPLNAFMLYMKEMRAKVIAECTLKESAAINQILGR 296
zebrafish       FDRSIYNKSHAEAKREKEPKKPVIKKPLNAFMLYMKEMRAKVIAECTLKESAAINQILGR 273
                **     *.*.*:***:*******************************************

channel         RWHALTREEQAKYYELARKERQLHMQLYPSWSARDNY----GKKKRRKRDKQQDSAT--- 349
zebrafish       RWHALTREEQAKYYELARKERQLHVQLYPSWSARDNYVSALGKKKRRKRDKQQDSSTGPG 333
                ************************:************    **************:*

channel         ----------------------
zebrafish       SPKKCRARFGLNQQTDWCGPCR 355
```

Supplementary Tables

Supplementary Table 1. Sequence inputs for the channel catfish reference genome assembly.

Library	Insert size (bp)	Average read length (bp)	Number reads	Sequence length (Mb)	Genome coverage	Usage
PCR-free paired end	400	118	749.5 M	88,627	88 X	Assembly
3kb mate pairs	3,000	102	17.8 M	1,834	53X clonal	Scaffolding
8kb mate pairs	7,800	103	4.7 M	484	36X clonal	Scaffolding
34 kb fosmid pairs	34,500	95	1.6 M	157	54X clonal	Scaffolding
PacBio	8-10 kb	1,637	3.3 M	5,424	5.4 X	Gap filling

Supplementary Table 2. Improvement of assembly contiguity with PCR-Free PE libraries and gap closing with Illumina and PacBio sequences.

	PCR-based PE libraries		PCR-Free PE library	
	Pre-gap close	Post-gap close	Pre-gap close	Post-gap close
Total number of gaps	91,749	46,528	85,860	24,025
Total gap length (bp)	28.3M	23.7M	16.0M	11.6M
Mean gap length (bp)	312	509	186	483
Scaffolds with gaps	7,475	7,372	1,726	1,429
Mean Scaffold Length (bp)	20,008	19,932	37,112	36,869
Mean Contig Length (bp)	6,136	9,623	7,235	17,085
Contig L50 (bp)	14,213	30,290	17,423	76,761
Contig L95 (bp)	1,611	2,246	2,366	7,742
Contig L99 (bp)	563	623	654	966

Statistics reflect automated assembly prior to manual scaffold correction.

Supplementary Table 3. Scaffolding using BAC end sequences (BES) and full length transcripts.

BES	Total number of BES	63,388
	Number of BES pairs	25,677
	Number of BES uniquely mapped to repeat-masked reference sequence	7,725
	BES uniquely mapped to same scaffolds	7,471
	BES uniquely mapped to different scaffolds	254
	Number of the scaffold pairs with at least two pairs of BES matches	39
	Number of scaffolds reduced by BES	21
Gene transcripts	Number of cDNAs that match ⩾2 scaffolds	94
	Number of scaffolds reduced by using cDNA	136

Supplementary Table 4. Definition of channel catfish chromosomes based on aggregate length of scaffolds in genetic linkage group (LG). Statistics do not include the inter-scaffold gaps.

Chromo-some	# of scaffolds	Physical size	# of genes	LG number	Genetic size (cM)	Average kb/cM	Average gene/Mb
1	25	37,506,655	1,233	1	139.5	268.9	32.9
2	45	37,241,186	1,295	3	169.7	219.5	34.8
3	32	34,901,054	1,091	6	163.3	213.7	31.3
4	39	34,498,793	990	4	128.1	269.3	28.7
5	41	32,117,789	1,137	11	152.7	210.3	35.4
6	10	32,336,212	914	17	138.2	234.0	28.3
7	45	32,227,861	1,152	8	117.3	274.7	35.7
8	20	30,408,258	966	9	146.9	207.0	31.8
9	15	30,148,133	986	16	145.3	207.5	32.7
10	19	29,044,225	907	20	126.0	230.5	31.2
11	12	28,206,976	801	7	102.1	276.3	28.4
12	13	28,144,356	1,007	12	104.8	268.6	35.8
13	21	27,389,953	943	5	110.2	248.5	34.4
14	31	26,696,778	847	18	114.8	232.6	31.7
15	13	26,074,281	932	15	115.7	225.4	35.7
16	10	25,964,284	843	25	127.6	203.5	32.5
17	30	25,913,994	834	22	68.4	378.9	32.2
18	11	24,511,808	911	13	131.1	187.0	37.2
19	39	23,654,134	744	23	93.3	253.5	31.5
20	14	21,426,798	799	2	116.2	184.4	37.3
21	16	20,985,506	713	21	110.3	190.3	34.0
22	22	19,855,691	693	26	101.2	196.2	34.9
23	4	18,814,537	658	19	118.9	158.2	35.0
24	15	19,557,860	734	24	106.9	183.0	37.5
25	10	19,456,751	679	28	114.1	170.5	34.9
26	27	19,063,806	744	10	105.8	180.2	39.0
27	14	18,540,812	553	27	113.7	163.1	29.8
28	22	18,160,844	750	14	113.8	159.6	41.3
29	19	15,253,832	525	29	109.5	139.3	34.4
Assigned	634	758,103,167	25,381	-	3505.4	218.4	33.8
Unassigned	9,340	25,091,658	1,280	-	-	-	-
Total	9,974	783,194,825	26,661				

Supplementary Table 5. Assessing the completeness of the channel catfish reference genome. The completeness of the channel catfish reference genome was assessed by *in silico* mapping of 167X genome coverage of re-sequencing reads to the reference genome.

Mapping statistics	Number of contigs	% contigs	% Sequence length
Mapped to scaffolds	483,512	93.9	99.00
Mapped to degenerates	11,250	2.2	0.37
Total mapped	494,762	96.1	99.37
Unmapped contigs	20,289	3.9	0.63
Total contigs	515,051	100.0	100.00

Supplementary Table 6. Assessment of the completeness of the channel catfish reference genome sequence through gene content contained in the sequenced fish genomes.

Species	# unique genes	Catfish+*	Catfish-§	Zebrafish+**	Zebrafish-§§
**Catfish**	26,661	-	-	723	970
**Zebrafish**	26,415	970	723	-	-
**Cave fish**	23,042	1,178	373	791	270
**Atlantic cod**	20,083	1,805	675	1,204	603
**Fugu**	18,518	1,923	198	1,375	132
**Medaka**	19,682	1,812	919	1,330	868
**Platyfish**	20,379	1,437	384	923	276
**Sole**	21,485	1,205	533	772	482
**Spotted gar**	18,341	1,272	463	832	397
**Stickleback**	20,785	1,719	1,042	1,134	981
**Tetraodon**	19,595	2,095	358	1,485	285
**Tilapia**	21,437	1,600	309	1,112	254
**Amazon molly**	23,614	1,210	463	786	431

*Catfish+ indicates the number of genes found in channel catfish but not from the species under comparison;

§ Catfish- indicates the number of genes not found in channel catfish but found from the species under comparison.

**Zebrafish+ indicates the number of genes found in zebrafish but not from the species under comparison;
§§ Zebrafish- indicates the number of genes not found in zebrafish but found from the species under comparison;

Supplementary Table 7. Comparative Analysis for orthologous chromosomes between channel catfish and zebrafish using single copy genes.
Z1 to Z25 represent zebrafish chromosomes, C1 to C29 represent catfish chromosomes. The number in the cell is the percentage of catfish genes on a chromosome that are homologous to zebrafish genes on the corresponding zebrafish chromosome. Percentages >5% are highlighted in yellow.

	Z1	Z2	Z3	Z4	Z5	Z6	Z7	Z8	Z9	Z10	Z11	Z12	Z13	Z14	Z15	Z16	Z17	Z18	Z19	Z20	Z21	Z22	Z23	Z24	Z25
C26	83	1	1	1	1	1	0	0	4	0	1	0	0	1	1	0	0	0	1	0	0	0	0	0	1
C29	53	1	1	0	0	1	19	1	1	2	1	0	11	1	0	1	0	0	0	1	0	3	1	0	0
C20	0	59	1	1	1	1	0	1	1	1	0	0	0	0	18	7	0	0	0	0	0	1	0	3	1
C2	0	1	57	1	0	1	1	1	1	0	1	1	0	6	0	1	0	0	1	24	0	0	0	1	0
C19	0	0	0	78	1	0	1	0	1	1	0	0	0	1	0	1	0	1	3	0	0	1	0	0	3
C28	0	1	1	0	85	0	1	1	0	0	0	0	0	1	1	0	0	0	0	0	0	1	0	1	1
C22	0	0	0	0	88	0	2	1	0	1	0	0	0	0	0	0	0	1	1	0	2	0	0	0	0
C11	2	1	1	1	1	81	1	0	1	0	0	0	0	0	1	0	0	0	0	0	1	2	2	1	0
C12	0	0	18	1	0	35	1	0	2	0	0	0	0	0	0	3	0	0	32	1	1	1	0	1	0
C7	1	42	1	1	1	1	46	1	0	0	0	0	0	0	1	0	0	0	1	1	1	1	1	0	0
C27	0	1	1	0	1	1	54	0	1	1	8	0	0	0	0	1	0	1	0	0	0	26	1	0	2
C5	0	1	1	1	0	1	0	85	1	0	1	0	0	0	0	0	1	0	0	0	1	1	1	0	1
C6	2	1	1	1	1	2	1	0	84	0	0	0	0	0	0	0	0	0	0	1	0	1	1	1	0
C16	1	1	0	1	1	1	1	1	0	87	1	0	0	1	1	0	0	1	0	1	0	0	0	0	0
C21	0	0	0	0	0	1	1	2	0	0	66	0	0	0	0	0	0	0	1	0	0	23	2	0	0
C13	0	0	3	0	0	0	0	0	0	0	0	88	2	0	0	0	2	0	0	0	0	0	0	0	0
C3	17	0	0	0	0	1	0	0	0	0	0	1	66	0	0	0	3	4	0	0	0	0	0	0	0
C8	1	0	0	1	0	0	1	0	0	0	0	0	0	63	0	0	0	0	0	0	1	0	0	0	26
C17	0	10	0	1	1	1	0	1	0	2	0	0	0	0	78	1	0	0	0	0	1	0	0	0	0
C1	0	2	1	0	0	1	0	0	0	0	0	0	0	0	1	58	0	0	3	0	0	1	0	29	0
C9	0	0	0	0	0	0	0	0	0	1	0	1	1	0	0	0	83	0	2	4	0	0	0	0	0
C4	0	0	0	2	1	1	1	0	1	1	1	0	2	1	0	0	0	84	0	0	0	1	0	0	1
C24	0	1	31	0	0	1	0	0	0	0	0	0	0	0	0	2	0	0	60	1	0	0	0	0	0
C25	0	1	1	1	0	0	1	1	0	0	0	0	1	0	0	1	2	1	0	86	0	2	0	0	0
C18	1	0	0	0	1	1	1	1	1	0	1	0	0	1	1	1	1	1	0	0	85	0	0	0	0
C10	0	2	1	1	0	1	29	0	1	0	26	0	1	0	0	0	0	1	0	0	0	30	1	0	1
C15	1	2	1	1	0	1	0	0	0	0	2	0	0	0	1	1	1	0	0	1	0	0	85	1	0
C23	0	1	0	0	0	0	0	0	0	0	1	0	0	0	0	40	0	0	2	0	0	0	0	49	0
C14	0	0	0	10	0	0	2	0	0	1	0	0	1	15	1	1	1	1	8	0	1	0	0	1	51

Supplementary Table 8. Comparative analysis of channel catfish and zebrafish genes.

Relationship type	Catfish	Core relationship	Zebrafish	Ratio
**One to one**	7,787	7,787	7,787	1:1
**One to two**	1,137	1,137	2,274	1:2
**One to many**	291	291	1,410	1:4.85
**Two to one**	1,924	962	962	2:1
**Two to two**	2,280	1,140	2,280	1:1
**Two to many**	822	411	1,527	1:1.86
**Many to one**	1,215	292	292	4.16:1
**Many to two**	1,674	408	816	2.05:1
**Many to many**	8,521	1,068	8,136	1.05:1
**Subtotal orthologs**	25,651	13,496	25,484	1.01:1
**In-paralogs**	143 (51 clusters)		243 (70 clusters)	
**Single copy species-specific genes**	867	-	688	-
**Total coding-gene**	26,661	-	26,415	-

Supplementary Table 9. Comparison of duplicated genes among zebrafish, fugu and channel catfish.

Species	Duplicated clusters	Total genes	Average copy number
Catfish	3,688	19,435	5.3
Fugu	3,262	12,673	3.9
Zebrafish	4,255	20,831	4.9

Supplementary Table 10. Genes commonly shared by all sequenced teleost fish species but absent from cartilaginous fish and jawless species, with high levels of expression in the swimbladder.

The gene expression levels in various tissues were determined in RPKM by RNA-Seq analysis of publicly available datasets: swimbladder (ERR023143 and ERR023148), skin (SRP013931), intestine (SRR1562529), and liver (SRR891504). The mRNA sequences from the total of 26,459 zebrafish genes were used as reference sequences for RNA-Seq analysis using CLC Genomics Workbench with default settings. The mRNA sequences were retrieved from Ensembl database (v78); only the longest transcripts were selected from each of those genes had isoforms.

Zebrafish Ensembl Gene ID	Gene Name	Swimbladder		Skin		Intestine		Liver	
		Reads	RPKM	Reads	RPKM	Reads	RPKM	Reads	RPKM
ENSDARG00000071014	s100u	4310	205.6	28110	39.6	1234	20.7	92	0.7
ENSDARG00000097712	si:ch1073-443n13.2	195	18.3	1287	3.6	36	1.2	31	0.5
ENSDARG00000094977	si:ch211-156j22.4	319	12.0	3356	3.7	51	0.7	17	0.1
ENSDARG00000092856	si:ch211-210c8.7	179	22.2	2332	8.5	52	2.3	106	2.1
ENSDARG00000086317	si:ch211-67e16.4	239	11.7	2459	3.6	107	1.8	92	0.7
ENSDARG00000070972	si:ch211-81a5.8	62	6.2	88	0.3	1	0.04	0	0
ENSDARG00000094422	si:dkey-12l12.1	8295	634.9	15433	34.9	4	0.1	1	0.01
ENSDARG00000076972	si:dkey-208k22.3	3730	175.8	12630	17.6	425	7.0	23	0.2
ENSDARG00000092123	si:dkey-20d21.12	143	13.2	946	2.6	2	0.07	14	0.2
ENSDARG00000093997	si:dkey-9i23.15	1107	87.4	8303	19.4	357	9.9	116	1.4
ENSDARG00000077303	zgc:174863	5315	304.3	36858	62.3	2557	51.4	322	2.9

Supplementary Table 11. A list of seven genes that are both present and expressed in pleco, but are not expressed in channel catfish.

**Accession**	**Gene description**
NP_001070846.1	Nuclear-interacting partner of ALK
XP_001339495.3	Protein FAM118B-like
NP_001138712.1	Secretory calcium-binding phosphoprotein 1 precursor
NP_001138708.1	Secretory calcium-binding phosphoprotein 5
XP_002942726.1	Uncharacterized protein LOC100495238
XP_005452781.1	Uncharacterized protein LOC102077212
XP_005460634.1	Uncharacterized protein LOC102080148

Supplementary Table 12. Primers used for analysis of SCPP gene expression in zebrafish.

Gene	Forward (5’ to 3’)	Reverse (5’ to 3’)
**SPARCL1**	AAGATGCAGAGTGACCAGGC	TGGATTCATCCCCACCAAGC
**SPP1**	GCCTCCATCATCATCGTAA	CCGTCTGTCGTCTAACTC
**SCPP1**	GCTCTGAATCACTTGAATCTG	TGCTGTTACTGTTGCTGTA
**ODAM**	CCTGTACAGCTGATGCCCAA	CAGGCACAGACGTTGCTTTC
**SCPP5**	AAGTACTCGCTCCCCAAAGC	AGGTGGCTGTTGAGGAACTG
**SCPP6**	AGACCACAACAGGGGCAAAA	ACGGCCCATGTTCTGAAGTT
**SCPP7**	TCGTCATTGGAGTTGCTTA	GCTGAGGAATCTCGTGTT
**SCPP8**	TCTATCACTGGACAAGAACA	AATCATCAAGCATTCGGAAG
**SCPP9**	GGTGCTGCGGTTATAGGACA	ATACGGCAAGCCCCCATTAG
**FA93E10**	TGACAGTGGCAGTAATGAG	AGCAACAGCACCATATCC
**GSP37**	CCACAACGAGCACAACAT	GAGCAGTGAAGAGATGATACA
**RPL13a**	TCTGGAGGACTGTAAGAGGTATGC	AGACGCACAATCTTGAGAGCAG
